# Deep neural network for detecting arbitrary precision peptide features through attention based segmentation

**DOI:** 10.1038/s41598-021-97669-7

**Published:** 2021-09-14

**Authors:** Fatema Tuz Zohora, M. Ziaur Rahman, Ngoc Hieu Tran, Lei Xin, Baozhen Shan, Ming Li

**Affiliations:** 1 David R. Cheriton School of Computer Science, University of Waterloo, Waterloo, ON N2L 3G1 Canada; 2 Bioinformatics Solutions Inc., Waterloo, ON N2L 6J2 Canada

**Keywords:** Computational biology and bioinformatics, Machine learning

## Abstract

A promising technique of discovering disease biomarkers is to measure the relative protein abundance in multiple biofluid samples through liquid chromatography with tandem mass spectrometry (LC-MS/MS) based quantitative proteomics. The key step involves peptide feature detection in the LC-MS map, along with its charge and intensity. Existing heuristic algorithms suffer from inaccurate parameters and human errors. As a solution, we propose PointIso, the first point cloud based arbitrary-precision deep learning network to address this problem. It consists of attention based scanning step for segmenting the multi-isotopic pattern of 3D peptide features along with the charge, and a sequence classification step for grouping those isotopes into potential peptide features. PointIso achieves 98% detection of high-quality MS/MS identified peptide features in a benchmark dataset. Next, the model is adapted for handling the additional ‘ion mobility’ dimension and achieves 4% higher detection than existing algorithms on the human proteome dataset. Besides contributing to the proteomics study, our novel segmentation technique should serve the general object detection domain as well.

## Introduction

Deep learning has exhibited unprecedented performance in a wide range of research areas, including the core machine learning problems, e.g., image processing, pattern recognition, natural language processing^1^, as well as, many multidisciplinary sectors, like bioinformatics, autonomous driving, fraud detection, etc. Proteomics is the large scale study of proteins—main workhorses responsible for biological functions and activities in a cell, tissue, or organism. Liquid chromatography coupled with tandem mass spectrometry (LC-MS/MS) based analysis leads the pathway of disease biomarker identification^2^, antibody sequencing^3^, neoantigen detection^4^, drug discovery and many other clinical research^5^. Most of these problems involve pattern recognition and sequence prediction, and thus the popular deep learning models (e.g., convolutional neural network (CNN), recurrent neural network (RNN), etc.) are worth applying in the proteomics domain as well. Bulik-Sullivan et al.^4^ proposed a deep learning based model EDGE, to develop neoantigen-targeted immunotherapies for cancer patients. Tran et al.^6^ proposed DeepNovo-DIA, a deep learning based de novo peptide sequencing technique, and later applied it on individual immunopeptidomes for personalized cancer vaccines^7^. AlphaFold^8^, developed by Google’s DeepMind, makes significant progress on protein folding, one of the core challenges in biology. Peptide feature detection from LC-MS map is a crucial step in the downstream workflow of protein quantitation and biomarker discovery. We proposed DeepIso^9^, the first deep learning model which combines recent advances in CNN and RNN to detect peptide features of various charge states and estimates their intensity. However, it is a fixed precision model (up to 2 decimal places), comparatively slower than other competitive tools, and incapable to adapt with higher dimensional data. But it gave us a good insight into the scope of deep learning in this context. So we make significant improvements in DeepIso and propose PointIso that resolves all those shortages.

**Figure 1:**
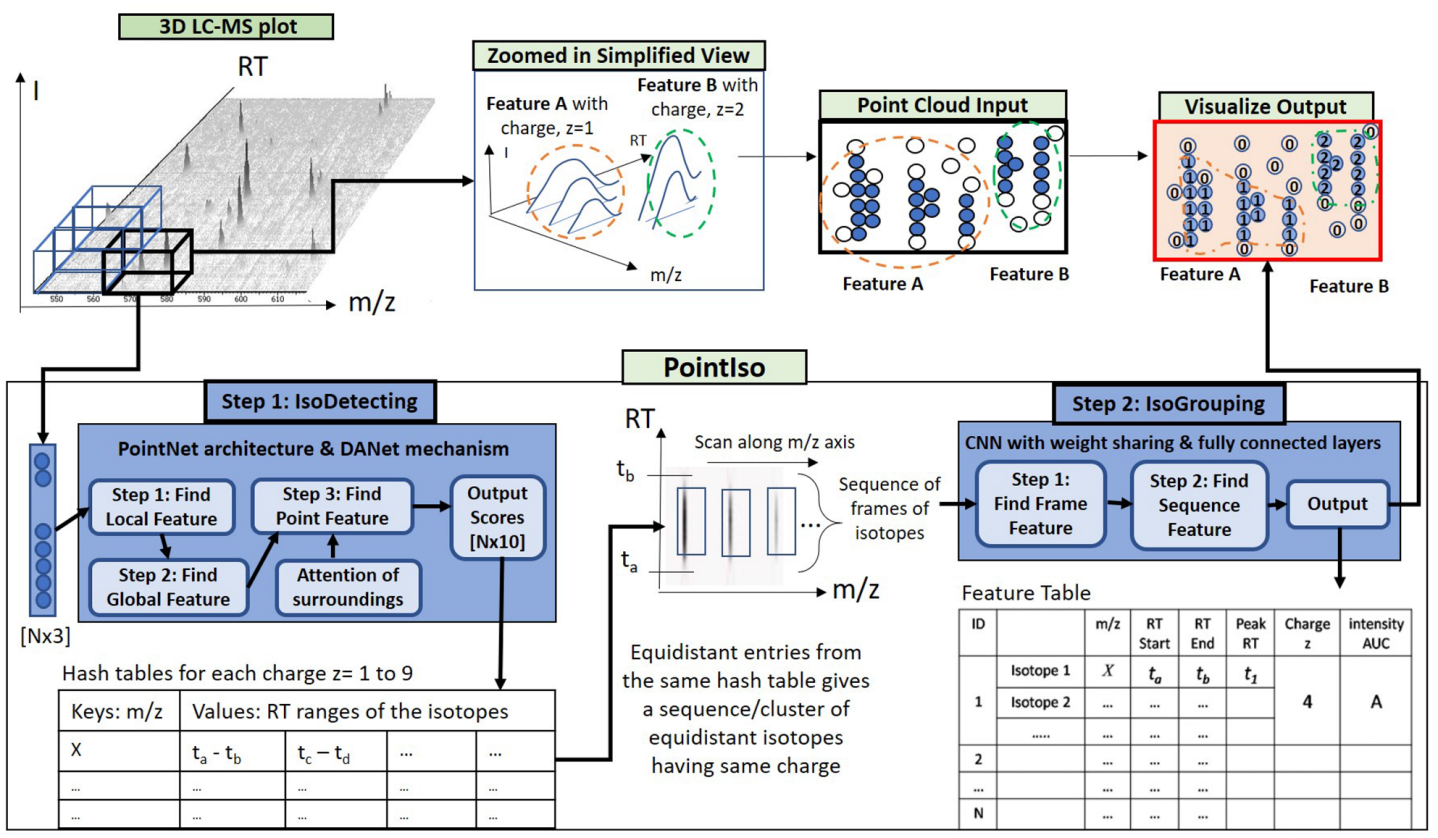
The workflow of our proposed model PointIso to detect peptide features from LC-MS map of protein sample. In 3D LC-MS plot we show a random scanning window in bold black boundary, enclosing two features. This region is further shown in the next image, labeled as ‘Zoomed in Simplified View’. Here, two features A and B are shown using orange and green boundary. The corresponding point cloud version of this window is shown in the next image, labeled as ‘Point Cloud Input’. Here the blue and white points correspond to the features and background points respectively. The ‘Visualize Output’ shows the PointIso predicted labels for the datapoints in that window. The datapoints labeled as ‘1’ belong to feature A having charge 1. And the datapoints labeled as ‘2’ belong to feature B having charge 2. The background or noisy datapoints are labeled ‘0’.

Many diseases are fundamentally linked to proteins. Therefore, if we can measure the relative protein abundance between the biofluid samples from a healthy person and disease afflicted person, we can identify the proteins which are either diagnostic or prognostic of the disease. Such proteins are called disease biomarkers. The LC-MS/MS based analysis is the current state-of-the-art technology for protein identification and quantification^10^. The procedure starts with digesting the protein into smaller peptides by various sequence-specific enzyme, and then the protein sample is passed to the first mass spectrometer (MS), where the peptides are ionized. The output of the first MS is called LC-MS map or MS1 data, which contains the three-dimensional peptide features as shown in Fig. 1. The three dimensions are mass-to-charge (*m*/*z* or Th), retention time (RT), and intensity (I) of peptide ions in that sample. Each peptide feature consists of multiple isotopes and appears during its elution time (RT range) on the map. The precursor ions (peak intensity isotope in the feature) are further passed to the second MS which generates MS/MS fragmentation spectrum that facilitates the identification of peptide sequence^11^, i.e., the amino acid sequence. We have to find the precise peptide feature boundary from LC-MS map, since this is a crucial step for many downstream workflows, e.g., protein quantification, identification of chimeric spectra, biomarker identification, etc.

There have been past attempts of peptide feature detection using several heuristic algorithms whose parameters are set by domain experts through rigorous experiments. Its prone to human error, since different settings result in significantly different outcomes. Some works apply machine learning, but involve a high level of feature engineering and human intervention instead of using the power of deep neural network to automate the major steps, e.g., detecting the features, separating the overlapping and adjacent features, and at the same time filtering out the actual features from noisy signals. Therefore, our target problem is to build up an automated system that performs these key steps by learning the parameters itself using the power of deep learning through several layers of neurons by training on an appropriate dataset. To be specific, we propose a deep learning based model that detects the peptide feature boundary (in three and four dimensional space) along with its charge state. Although this resemblance common pattern recognition problems in machine learning, however, there are several reasons what make the peptide feature detection far more challenging than the general cases. For instance, frequent overlapping among the multi-isotope patterns, not always obeying typical conventions, smooth blending of features with noisy signals, and hundreds of thousands of peptide features which are comparatively tiny in size as compared to the massive background (RT axis span over 0–120 min, and *m*/*z* axis ranges from 400 to 2000 *m*/*z* with resolution as high as up to 4 decimal places). DeepIso^9^ shows better performance than other existing heuristics based tools. However, it cannot accept high-resolution input due to using image-based CNN, and comparatively slower than other competitive tools because of using classification network in an overlapping sliding window approach for doing the feature segmentation. Therefore, we bring significant changes to overcome these problems and offer PointIso that gives precise boundary information in a time-efficient manner and achieves a higher percentage of feature detection. In particular, we change the image based classification network to a point cloud based segmentation network. The point cloud is a data structure for representing objects using points, e.g., using triplets in a three-dimensional environment. We combine point cloud based deep neural network PointNet^12^ and Dual Attention Network (DANet)^13^ to integrate local features with their global dependencies and some context information. Unlike DeepIso where 2D projected images are used for representing 3D peptide features, we adapt PointNet to our context in order to directly process the 3D features. It makes it feasible to accept input data with two or more times higher resolution (arbitrary-precision) than DeepIso and achieves better detection. On the other hand, the original DANet is proposed for finding the correlated objects in the input landscape image for the autonomous driving problem. We take the idea and plug it into the PointNet network to solve boundary value problems during scanning the huge LC-MS map through *non-overlapping* sliding windows, which makes it three times faster than DeepIso. Another important contribution of this paper is to adapt this 3D model to handle the additional dimension introduced by the latest technology, namely the ‘trapped ion mobility’ spectrometry with Time-of-flight instrument (TimsTOF). It adds ion mobility ($\frac{1}{k0}$) as another dimension of separation^14^. Such instruments offer several desirable properties for the analysis of complex peptide mixtures and becoming popular in shotgun proteomics, e.g., analyzing immune suppression in the early stage of COVID-19 disease^15^. Therefore, we show how to adapt our original 3D model to handle the additional axis information, i.e., 4D LC-MS data. We believe this adaptation capability of our PointIso model with multiple contexts should make it more appealing in the proteomic society. Besides that, our novel concept of attention based scanning of LC-MS map through a completely non-overlapping sliding window also has the potential to serve the general image processing problems. Therefore, we believe PointIso makes a notable contribution in accelerating the progress of deep learning in proteomics area, as well as, general pattern recognition study.

## Results

We explain the intuition of our proposed model using the workflow shown in Fig. 1. We see the three-dimensional LC-MS map in the upper left corner and PointIso starts with scanning this map by sliding a window along two directions: *m*/*z* axis and RT axis. The third axis tracks the signal intensity, I. A sliding window (or a target window) is essentially a 3D cube of point cloud. We also see that PointIso model works through two modules, IsoDetecting in the first step, and IsoGrouping in the second step. So the point cloud input consists of a set of ‘N’ datapoints which is passed as input to the IsoDetecting module as shown by the arrow sign from the sliding 3D window. IsoDetecting module segments the datapoints as z = 0–9, where z = 0 means the respective datapoint belongs to noise or background, and z = 1–9 means the respective datapoint belongs to a feature having charge z. We build this module by incorporating the attention mechanism offered by DANet into the PointNet architecture, to support *non-overlapping* sliding windows. The IsoDetecting module produces a list of isotopes of potential features that is recorded in a hash table. Then in the second step, IsoGrouping module takes those sequences of isotopes (each sequence may have any number of isotopes) and predicts the boundary (first and last isotope) of features. Each sequence may be broken into multiple features or merely predicted as noisy signals. This prediction finally gives us a feature table that reports the detected peptide features along with the monoisotopic *m*/*z* (the first isotope of a feature), charge, RT range of each isotope, and intensity. We can also visualize the final result as shown in the image labeled as ‘Visualize Output’ in Fig. 1 (upper right corner).

We downloaded the benchmark dataset from ProteomeXchange (PXD001091) that was prepared by Chawade et al.^16^ for data-dependent acquisition (DDA). The samples consist of a long-range dilution series of synthetic peptides (115 peptides from potato and 158 peptides from human) spiked in a background of stable and nonvariable peptides, obtained from *Streptococcus pyogenes* strain SF370^17^. Synthetic peptides were spiked into the background at 12 different concentration points resulting in 12 samples, each having multiple replicates. We obtain an LC-MS map from each replicate, totaling 57 LC-MS maps for the experiment.

### Training of PointIso

Since we are using a supervised learning approach, we need labeled data for training. Human annotation of peptide features is out of scope due to the gigapixel size of the LC-MS maps^18^. Therefore, we match the feature lists produced by MaxQuant 1.6.3.3 and Dinosaur 1.1.3 with a tolerance of 10 ppm *m*/*z* and 0.03 min RT and take the intersection set as the training samples. In PointIso we also need the precise boundary information (i.e., RT time range and *m*/*z* value of each isotope of the features) which is not generated for the users in MaxQuant. Therefore we use Dinosaurs for that information. The IsoDetecting and IsoGrouping modules are trained separately using suitable training data. To generate training samples for the IsoDetecting module, we place a scanning window over the features and cut the region along with the surrounding area. The total number of features available for charge z = 1 to 9 are provided in the “Methods” section. The input resolution of our dataset is up to 4 decimal places along *m*/*z* axis (whereas DeepIso accepts only 2 digits after the decimal point). For training the IsoGrouping module, we cut a sequence of frames (each frame holding an isotopic trace) from these peptide features. Training data generation technique is explained in “Methods” section. We apply $k=2$ fold cross-validation^19^ technique to evaluate our proposed model which is elaborated in Supplementary Note E.

### Performance evaluation of PointIso

We run MASCOT 2.5.1 to generate the list of MS/MS identified peptides, where we consider the identifications with peptide score > 25 (ranges approximately from 0.01 to 150) as high confidence identifications^5^. For performance evaluation, we compare the percentage of high confidence MS/MS peptide identifications matched with the peptide feature list produced by our algorithm and some other popular algorithms. Since the identified peptides must exist in LC-MS maps, therefore, the more we detect features corresponding to them, the better the performance^5,18,20,21^. The other tools used for comparison are MaxQuant 1.6.17.0^22^, OpenMS 2.4.0^21^, Dinosaur 1.2.0^23^, and PEAKS Studio X^24^. We use the benchmark dataset prepared by Chawade et al.^16^, and follow the MaxQuant parameters published by them. For Dinosaur, default parameters mentioned at their GitHub repository (https://github.com/fickludd/dinosaur) are used. For OpenMS, we use the python binding pyOpenMS^21,25^ and follow the centroided technique explained in the documentation. For all of the feature detection algorithms, we set the range of charge state 1–9 (or the maximum charge supported by the tool).

**Figure 2:**
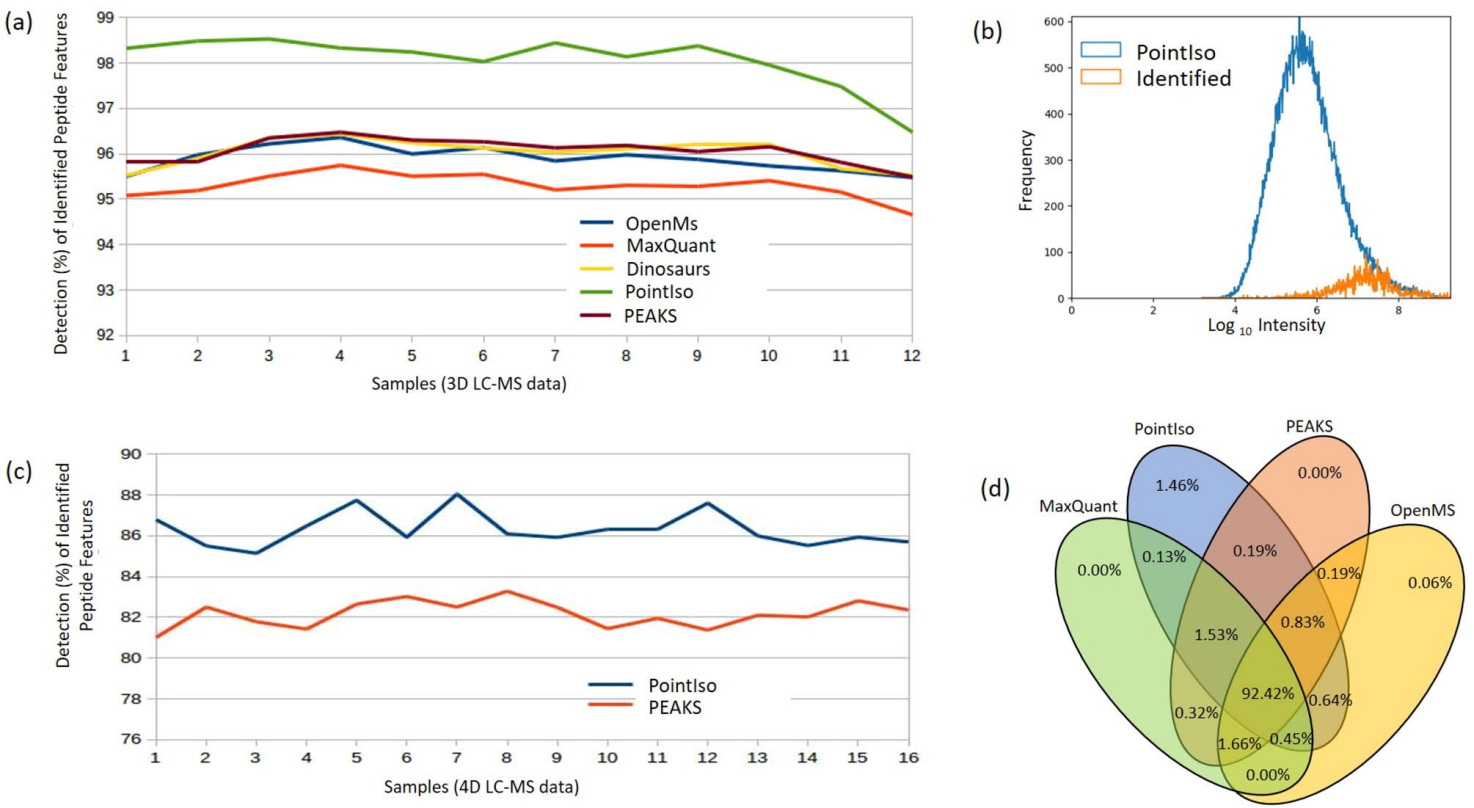
(**a**) Detection percentage of identified peptide features by different tools for 12 samples, each having different concentrations. (**b**) The intensity distribution of peptide features detected by PointIso (blue) and identified by database search (orange). We see that the distribution of identified features is wedged into the high-intensity tail of the distribution of detected peptide features because only high-intensity features are selected for fragmentation. (**c**) Detection percentage of identified 4D peptide features by PEAKS and PointIso for 16 samples generated by TimsTOF instrument (sample 1–12 belong to phase A and sample 13–16 belong to phase B). (**d**) Venn diagram of identified peptide features detected by different algorithms (we show four algorithms to keep the Venn diagram simple). We see that there are about 1.46% peptide features which are detected exclusively by PointIso. In the Supplementary Fig. S4 we show the type of features which are missed by other feature detection tools.

#### Percentage of identified peptide features detected by PointIso

We show the plot of detection percentage of high confidence MS/MS identifications with error tolerance of 0.01 *m*/*z* and 0.2 min peak RT^9,16,23^ for 12 samples by different algorithms in Fig. 2a. We see that PointIso has a significantly higher detection rate for all the samples. The average detection rate of PointIso is 98.01% presented in the first row of Table 1 (the entire result can be found in Supplementary Table S3). Then we match all the MS/MS identifications (any score) with the peptide features as presented in the second row. We see that our algorithm consistently provides a higher detection rate than other tools. Please note that multiple MS/MS fragments coming from different peptide features can match with the same peptide sequence during the database search. Unlike the experiments with DeepIso, here we treat those MS/MS identifications as different entities if their *m*/*z* and peak RT values are significantly different. We further emphasize the fact that, although the model is trained on sample features from certain concentration (e.g., sample 5, 6, 7, 8), it can detect features having higher or lower concentration as well (e.g., sample 1–4, and 9–12). It implies that the model can well generalize the peptide feature properties irrespective of peptide intensities seen during training time. Therefore, once we train a model on a protein sample, the same model should be applicable to other protein samples of a similar nature without further training, making it more appealing in the practical sectors.

**Table 1:** Percentage of MS/MS identifications matched by feature list produced by different algorithms.

Matching criteria	MaxQuant	OpenMS	Dinosaur	Peaks	DeepIso	PointIso
MS/MS identifications with high confidence score	95.24%	95.86%	96.01%	95.66%	96.05%	98.01%
All MS/MS identifications	93.73%	94.03%	94.90%	94.82%	94.10%	96.98%

**Figure 3:**
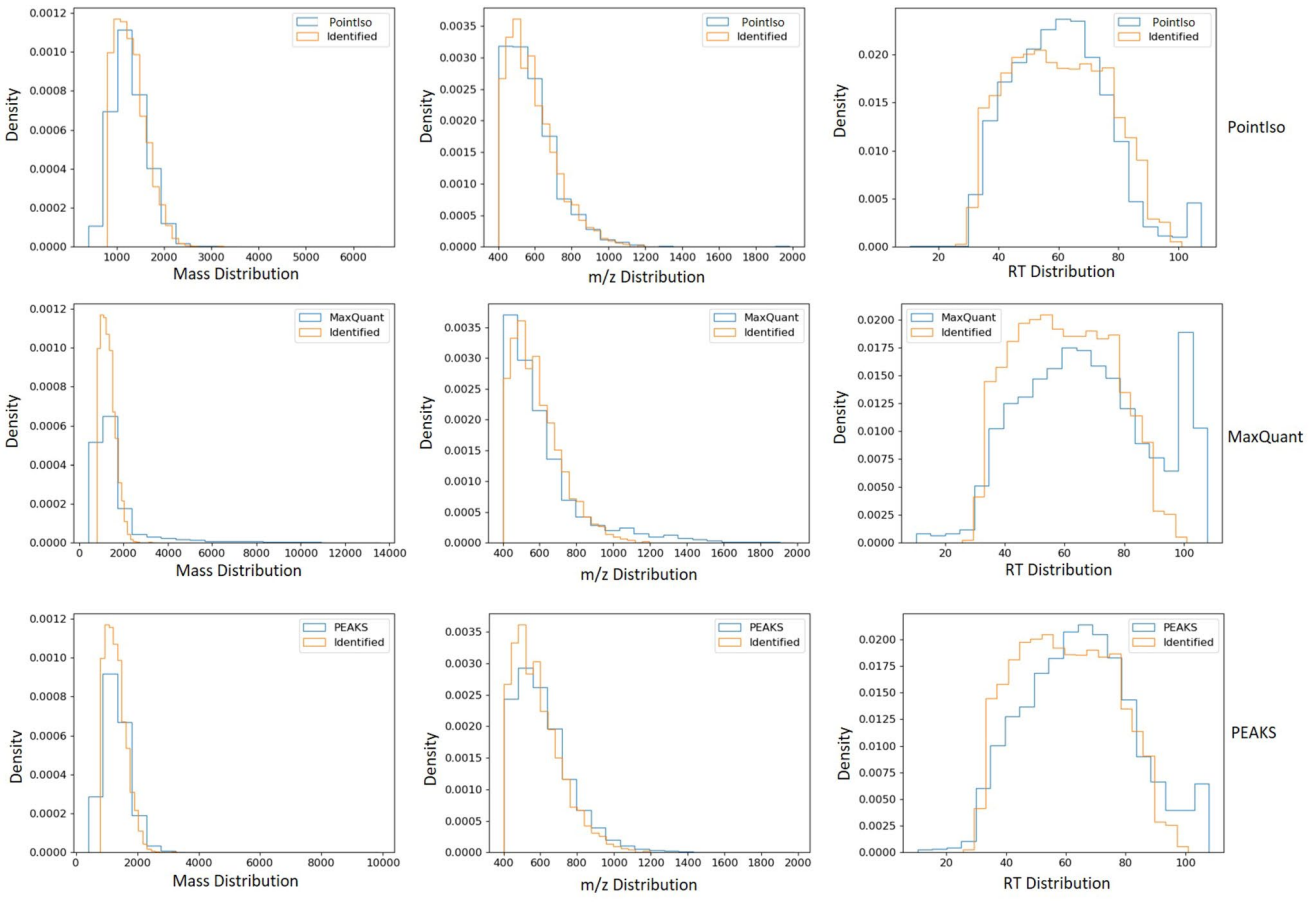
Comparison of mass, *m*/*z*, and RT distribution of detected features (blue) and identified features (orange) for different tools. First, second, and third rows of plots are representing the result for PointIso, MaxQuant, and PEAKS respectively (distributions for other tools are included in Supplementary Fig. S3). The orange distributions remain the same along the column since its representing the identified peptides. Plots in the first column show that all algorithms might have some false positives below 1000 Da mass, but the rate is lowest for PointIso. MaxQuant may have some false positives above 2000 Da as well. Then the second column is representing the distribution of *m*/*z*. Again, MaxQuant might have some false positives in the higher range of *m*/*z*. Finally, the third column is presenting the distribution of RT and all the tools have a probability of detecting false positives above 100 min RT, but that rate is lowest for PointIso. Besides that, both MaxQuant and PEAKS might report some false positives below 30 min RT. For PointIso, the histograms of detected features (blue) are showing good alignment with the histograms of identified features (orange) and support the correctness of the detected features.

#### Quality of peptide features detected by PointIso

Next, we discuss our observation on the peptide features detected by PointIso, but non-identified by database search. The total number of features reported by OpenMS, MaxQuant, Dinosaur, PEAKS, DeepIso, and PointIso is respectively 60, 000, 42, 000, 45, 000, 41, 000, 50, 000, and 100, 000 approximately. Because of high number of features reported by PointIso, we wanted to verify whether those features are reliable or not. The DDA mode selects the most abundant peptides for fragmentation and identification. As a result, some actual peptides having lower abundance remain unidentified. The real number of peptide species eluted in a single LC run can actually be over 100,000 as discovered by Michalski et al.^26^. During biomarker identification through relative protein quantification, it is desirable to assess the identity of those remaining peptide features which do not readily match with any MS/MS identification but shows significant abundance change among multiple samples. This is done by performing post-annotation of the remaining peptide features through targeted MS/MS^2^. That is why this is important to verify whether the PointIso detected but non-identified features are potential peptide features or not. The typical statistical methods for finding the false positive rate are not applicable here due to the absence of ground truth about the existence of those non-identified peptide features^9,18^. However, one reliable technique is to observe their intensity distribution which should be log-normal and if there are many false positives then there will be multiple peaks in the distribution^26^. We present the intensity distribution of the detected features by PointIso in an LC-MS/MS run in Fig. 2b which is quite well behaved. Besides this, we also investigated the physicochemical properties of the two populations (blue and orange) as shown in Fig. 3. It demonstrates that the PointIso detected peptide-like features have a good probability of being true peptides^26^. Therefore, we can state that PointIso reports reliable features besides having a high detection rate of identified peptides. We also report the percentage of identified peptide features that are exclusively detected by PointIso, in the Venn diagram of Fig. 2d. We visually observed some of those, and it appears that, merging the features having very close peaks along RT axis, missing monoisotopes, merging overlapping features, and broken signals, these are the main reasons of not detecting some features by other tools, although nicely detected by PointIso (illustrated in Supplementary Fig. S4).

#### Peptide feature intensity calculation by PointIso

The correctness of peptide feature intensity depends on whether the bell-shaped signals are detected nicely or not. The Pearson correlation coefficients of the peptide feature intensity (area under the isotopic signals of peptide feature) between PointIso and OpenMS, MaxQuant, Dinosaurs, PEAKS are respectively 93.76%, 95.31%, 89.88%, and 88.93%. Our algorithm has a good linear correlation with other existing algorithms, which validates the correctness of peptide feature boundary or area detection by our model.

#### Time requirement of PointIso

The total time of scanning the LC-MS map by IsoDetecting module and IsoGrouping module is the running time of PointIso model. We present the running time of different algorithms and the platforms in Table 2. PointIso model is about three times faster than DeepIso and has a comparable running time with most of the existing tools. PEAKS is much time-efficient than all other algorithms. However, we believe that the PointIso can be made faster as well, by using multiple powerful GPU machines in parallel.

**Table 2:** Approximated running time of different algorithms. Here the platform used for OpenMS, DeepIso and PointIso did not have support for running Windows application of PEAKS, MaxQuant, and Dinosaur. So we used different machine for running those.

Platform	Processor: Intel Core i7, 4 cores OS: Windows 10 for running the applications			Processor: Intel(R) Xeon(R) Gold 6134 CPU, NVIDIA Tesla OS: Ubuntu 16.04.5 LTS for running the python scripts		
Algorithms	PEAKS	Dinosaur	MaxQuant	PointIso	DeepIso	OpenMS
Running time	8 min	15 min	30 min	30 min	1 h and 40 min	2 h and 50 min

#### Adaptation of PointIso for higher dimensional data

Finally, we believe the generalizability of a model is a measure of the usefulness of that approach for a broader group of contexts. Therefore, we perform easy alteration of our PointIso model to support four-dimensional point cloud input (illustration is provided in “Methods” section), so that it can also analyze ion mobility dimension in TimsTOF generated dataset. For evaluation, we used the dataset containing human cervical cancer cells (HeLa) grown in Dulbecco’s modified Eagle’s medium with 10% fetal bovine serum, 20 mm glutamine, and 1% penicillin-streptomycin (all Life Technologies Ltd., Paisley, UK)^14^. In particular, we downloaded the HeLa raw files (16 samples) from ProteomeXchange (PXD010012) generated by the Evosep One instrument, a new high-performance liquid chromatography (HPLC) instrument employing an embedded gradient and capable of fast turnaround between analyses^27^, which is promising for rapid yet comprehensive analysis. For example, to analyze protein interactomes, or quantification of trace-level host cell proteins (HCPs) in recombinant biotherapeutics. The common set of feature lists produced by MaxQuant 1.6.3.3^28^ and PEAKS Studio X is used for the training like before (there is no other software performing peptide feature detection on TimsTOF data as per our knowledge). In the evaluation step, we used MSFragger-3.2, an ultrafast and comprehensive peptide identification tool in mass spectrometry-based proteomics^29^ that is free to use for academic purpose (our licensed MASCOT version does not have support for processing latest TimsTOF data). The MS/MS spectra were matched to human reference proteome (Uniprot, 2021/06, including isoforms) using the default closed search settings parameters. In the dataset, two mobile phases, A (12 samples) and B (4 samples), were water with 0.1% formic acid (v/v) and 80/20/0.1% ACN/water/formic acid (v/v/vol), respectively. With 0.01 $\frac{1}{k0}$, 0.01 *m*/*z*, and 0.2 min RT tolerance, peptide feature detection by PointIso and PEAKS are 86.31% and 82.16% respectively. The result for 16 samples are provided in Fig. 2c. The running time of PEAKS and PointIso for this 4D dataset is respectively about 25 min and 45 min. Therefore, we can say that PointIso is adaptable to diverse datasets implying greater utility.

## Discussion

We propose PointIso, a deep learning-based model that discovers the important characteristics of peptide features by proper training on a vast amount of available LC-MS data. Other heuristic algorithms have to set different parameters, e.g., the number of scans to be considered as a feature, centroiding parameters, theoretical formulas for grouping together the isotopes, and also data dependant parameters for noise removal and other preprocessing steps. On the other hand, PointIso does not rely on manual input of these parameters anymore and systematically learns all the necessary parameters itself, which is the main strength of this model. In this section, we will first demonstrate the justification of different design strategies performed. Then we will discuss the significant changes made in the PointIso model for the adaptation with TimsTOF generated 4D data. Finally, we will conclude by referring to some potential research directions.

**Table 3:** Performance of PointIso in different developmental stages (based on validation dataset).

Model	Matching with MS/MS identified peptides
Initial model	65%
Bi-directional 2D RNN	72%
Dual attention mechanism	94%
Fine tuning of IsoDetecting module with long RT range	95.5 %
Increasing resolution from 0.01 *m*/*z* to 0.0001 *m*/*z*	97%
Fine tuning using features detected with wrong charge by IsoDetecting module	98.22%
New architecture of IsoGrouping module	99.55%
Fine tuning with feature like noises	98.52%

We will first discuss why our point cloud based system is preferred over the image-based algorithm, e.g., DeepIso. The reason is twofold. First, we want to change the classification network (that slides scanning window pixel by pixel and generate prediction for each pixel) to a segmentation network that can predict all the datapoints at a time, making the process quite faster. Second, we want to accept higher resolution with arbitrary precision. Now, a scanning window covers 15 RT and 2.0 *m*/*z*. If we consider a resolution of up to four decimal points, 2D image based segmentation network will need to segment 300,000 pixels ($15 \times \frac{2.0}{0.0001} = 300,000$), where most of the points will be blank. However, with point cloud representation it has to predict the label of about 5000 points only. So, the 2D image representation of 3D features makes the segmentation problem unnecessarily voluminous, especially with higher resolution. Therefore, to support higher resolution compatibility and a faster speed, we move from image based classification network to point cloud based segmentation network. There are also other literature, e.g., PointNovo^30^, which switched to point cloud representation for supporting higher resolution data like us.

In PointIso, we have to deal with a highly class-imbalanced problem with this segmentation network of the IsoDetecting module. We use class weights (decided based on the class distribution per sample) while calculating cross-entropy loss so that both the positive and negative datapoints are learned well. More discussion and empirical results regarding this are included in Supplementary Note A. This initial model was able to achieve about 65% matching with the peptide identifications as shown in Table 3. Therefore we were in need of further investigations for the improvement which are discussed next.

**Figure 4:**
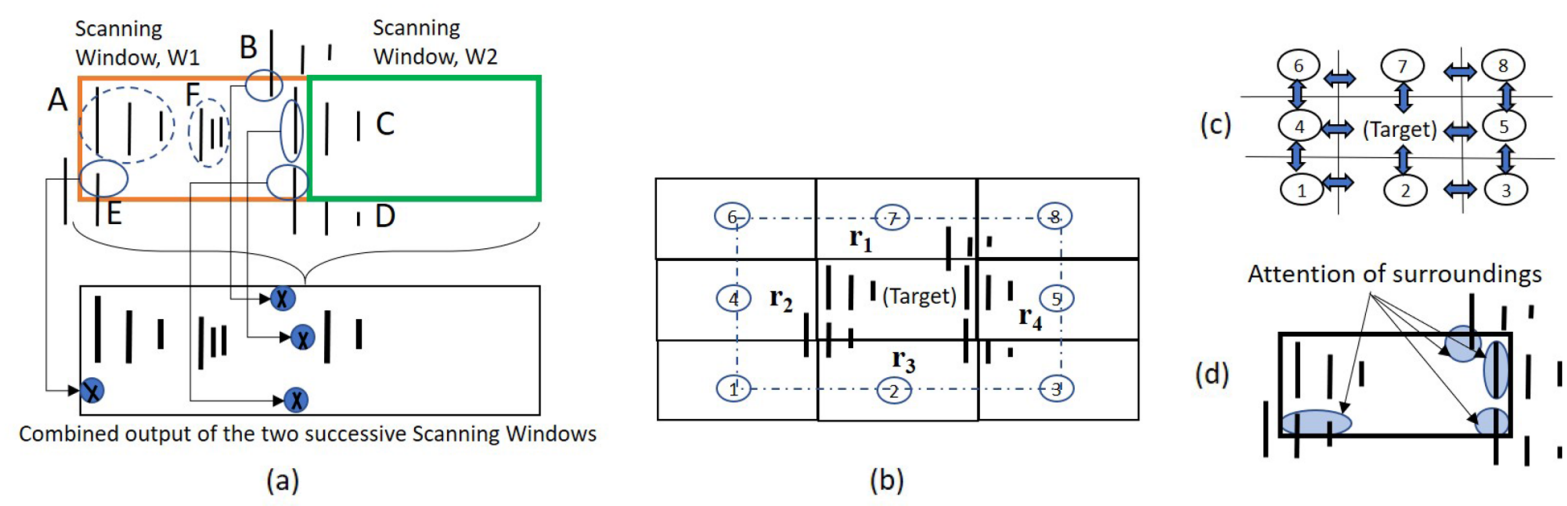
(**a**) We show two successive and non-overlapping scanning windows, W1 and W2, and six features: A, B, C, D, E, and F. We show the combined output of the two successive scanning windows in the bottom image. Feature A and F are fully contained within W1. Therefore, all of its isotopic signals are correctly detected, as shown in the combined output. However, for each of the other four features, W1 sees partial traces shown by small circles in the upper image. Without any background knowledge, those traces are not adequate for deciding whether they belong to real features or merely noisy traces. We mark the isotopes by a cross sign which are not detected. The second window W2 detects two isotopes of feature C. But the system missed the monoisotope of feature C, which is treated as missing the feature as a whole. (**b**) Surrounding regions of a target window. (**c**) 2D bi-directional RNN to flow the surrounding information towards the target window in center. (**d**) Attention of surrounding regions over the datapoints of target window.

**Figure 5:**
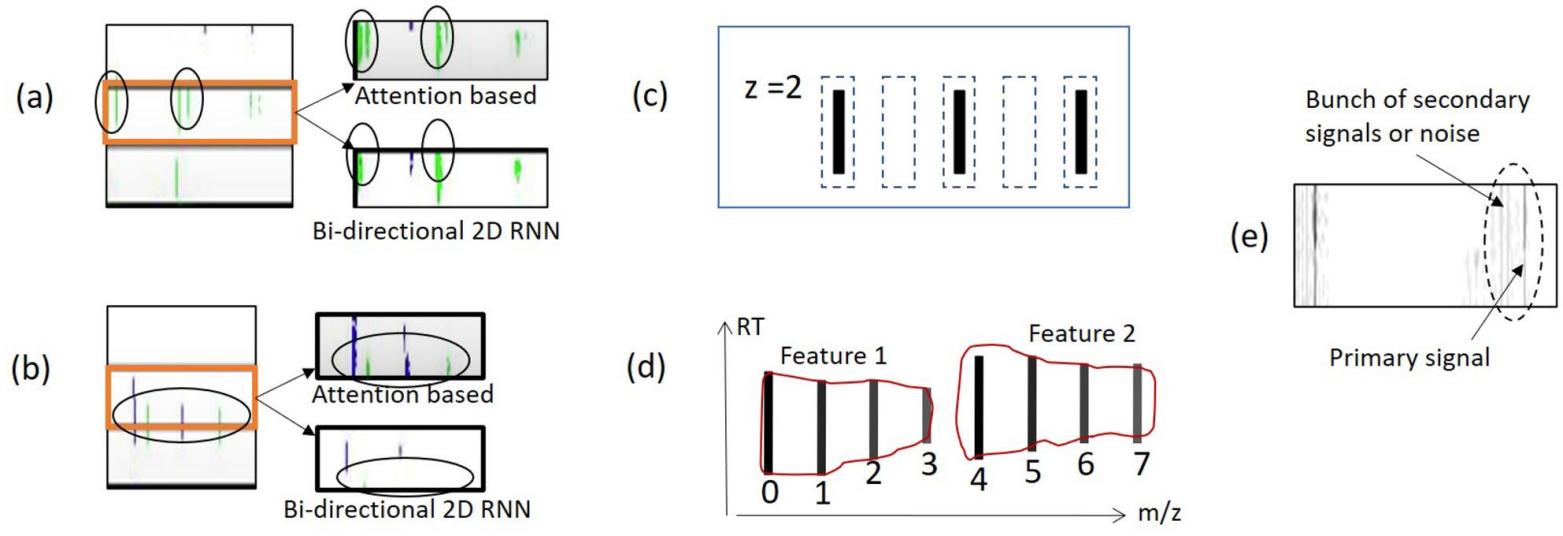
(**a**) and (**b**) are showing the comparison between attention-based mechanism and bi-directional 2D RNN. The orange rectangle is showing the target window. For each target window, detections by attention mechanism and bi-directional two-dimensional RNN are shown next to it, pointed by arrow signs. We see that the attention mechanism works better in separating closely residing features in (**a**) and detecting partially seen features in (**b**). (**c**) When isotope lists are passed to the IsoGrouping module with wrong frames (dotted rectangles) because of the wrong charge ($z=4$) detected by the IsoDetecting step, it results in discarding this whole group of frames as noise due to the inconsistency (blank frames) observed. (**d**) Adjacent feature problem. (**e**) Feature like noisy signals.

We would like to explain the reason for using the attention mechanism with the segmentation network of the IsoDetecting module. In a random scenario, features can spread over multiple windows. Applying a segmentation network without any surrounding knowledge will cause missing of the features, e.g., feature C, as illustrated in Fig. 4a. It also fails to compute the total abundance of features properly, since its missing trailing regions of features like B, D, and E. To overcome this problem, we have to incorporate surrounding knowledge while segmenting the datapoints of a target window, i.e., W1 in Fig. 4a. According to our experiments, we find that the regions $r_1$, $r_2$, $r_3$, and $r_4$ in Fig. 4b are actually playing the key role in detecting the traces inside target window. Just using a big window and predicting the smaller center region points does not solve the problem according to our experimental result. The results with other different criteria: 50% overlapping of scanning window, 2D bi-directional RNN (Fig. 4c), and the attention mechanism (Fig. 4d) inspired by DANet are presented in Supplementary Note B. Besides that, we also had a visual verification of whether the partially seen peptide features are properly detected or not as presented in Fig. 5a,b. Since the PointNet segmentation network combined with DANet works better than other techniques, we choose this strategy to develop our IsoDetecting module.

Next, we discuss how the IsoGrouping module supports the higher resolution output coming from the IsoDetecting module. IsoDetecting module outputs the isotope list with *m*/*z* resolution of up to 4 decimal places, but IsoGrouping module does not need that much high resolution to group together the potential isotopes into a feature. Therefore, we use a resolution degrading approach before passing the isotope lists to IsoGrouping module. The isotopes who merge in the lower resolution are kept in separate lists and thus passed to the IsoGrouping module separately. This resolves the problem of missing features merged in lower resolution. Besides that, we filter out the signal area from background while passing the frames (unlike DeepIso), based on the boundary information provided by IsoDetecting module, so that IsoGrouping module can see the bell curve better than before. As a result, we can keep the module simple by avoiding attention gate (which was required in DeepIso). Unlike DeepIso, which uses the RNN layer to process frames of the sequence one at a time, we process five frames at a time using a network consisting of CNN and fully connected layers through weight sharing. It results in better prediction at the output layer. Other new additions are: we incorporate area under the isotopic signal as context information through embedding (which reduces the uncertainty during class prediction) and feed the charge into the network through a scaling gate neuron, which helps in a proper grouping. The final architecture of IsoGrouping module is quite different than the one in DeepIso and improves the feature detection by about 1.5%, as presented in the seventh row of Table 3. More are presented later in the “Methods” section.

Fine tuning the primary model by feeding back the misclassified data played an essential role in overall improvement. Some examples include, features detected with wrong charge (Fig. 5c), adjacent features (Fig. 5d), secondary signals (Fig. 5e) etc., which are fed back to the model for further learning. This topic is elaborated in Supplementary Note C.

We believe that the good adaptation capability of a model towards various datatypes is one of the desired qualities to extend the practical utility. Therefore, we adapt our model to handle more advanced data type, namely TimsTOF generated data which has the additional ‘ion mobility’ dimension. To integrate mass spectrometry-based proteomics into biomedical research, and especially into clinical settings, high throughput and robustness are essential requirements. That is why we performed the experiments on the 4D LC-MS data generated by Evosep One instrument. The significant changes we had to make are in the IsoDetecting module since it has to segment 4D points now. The scanning window dimension covering 2.0 *m*/*z* and 15 min RT now also spans along the new $\frac{1}{k0}$ dimension. To keep the changes minimal, we let each scanning window cover the whole range of $\frac{1}{k0}$ (0 to about 1.5 with the resolution of up to 5 decimal points) so that we do not need to translate the scanning window along the new axis. However, this increases the number of datapoints by 3 times which is not feasible to load in GPU memory. That is why we reduce the scanning window dimension to 1.0 *m*/*z* and 10 min RT. Besides that, we read the $\frac{1}{k0}$ values every third datapoint in the ion mobility dimension. After making this alteration the number of datapoints within a scanning window is about 6000, which is loadable in our GPU memory. Next, in the IsoGrouping module, we avoid weight sharing among the five frames and different weights are learned for different frames. We believe the reason behind this is the higher complicity associated with the 4D patterns. Details are provided in the “Methods” section. We show a way of adaptation strategy that works well with the TimsTOF generated 4D data. We believe users can make simple modifications of the PointIso model in different ways based on the context and available GPU memory which makes PointIso generalizable to various domains, making it more appealing in the proteomic society.

Finally, we refer to the fact that, although we have prepared the training data taking the common set of feature lists reported by two existing algorithms, it does not imply that PointIso gains the same knowledge as those algorithms. The common set is used only to replace human annotators, but the outcome of PointIso is quite different from those algorithms. Because deep learning network learns the required parameters by stochastic gradient descent through several layers of neurons and back-propagating the prediction errors, a completely different technique than all the existing heuristic methods. That is why PointIso achieves a higher detection rate than others. Some appealing future works should involve plugging in PointIso in the downstream workflow of peptide quantification, identification of chimeric spectra, and disease biomarker identification. Extending this model for Intact Mass analysis might also be another important research direction. Also, it will be interesting to see how the attention based non-overlapping sliding window approach performs in general object segmentation problems. We are looking forward to these research opportunities in the future.

## Methods

Our model is trained and evaluated through two fold cross-validation. That is, we divide the LC-MS maps in the dataset into two groups or two folds. Then we train on one group and test on the other group, and vice versa. We discuss how the LC-MS maps are divided into different folds for cross-validation in Supplementary Note E. To save the best model state during training, we keep one LC-MS map from the training set as a validation set. Then we use that model state for testing. Whenever we say train or validation on an LC-MS map, we mean training/validation on the features cut from that LC-MS map (explained later under the subsections regarding training data generation for IsoDetecting and IsoGrouping module). However, when we say testing on an LC-MS map, we actually mean scanning the full LC-MS map as shown in the block diagram of Fig. 1. That is, during testing (or the real application phase) we will scan the whole LC-MS map in a bottom-up, left to right fashion (in other words, column by column). Our model runs the processing on raw LC-MS map which is obtained in .ms1 format using the ProteoWizerd 3.0.18171^31^. Then we read the file and convert it to point cloud based hash table where RT scans are used as keys and (*m*/*z*, intensity) are inserted in a sorted order under those keys. Therefore we have the datapoints saved as triplets (RT, *m*/*z*, intensity) in the hash table. In the following sections, we will discuss the scanning procedure of IsoDetecting and IsoGrouping modules, technical details on model training including training data generation, and finally, extension of the modules for four-dimensional data.

**Figure 6:**
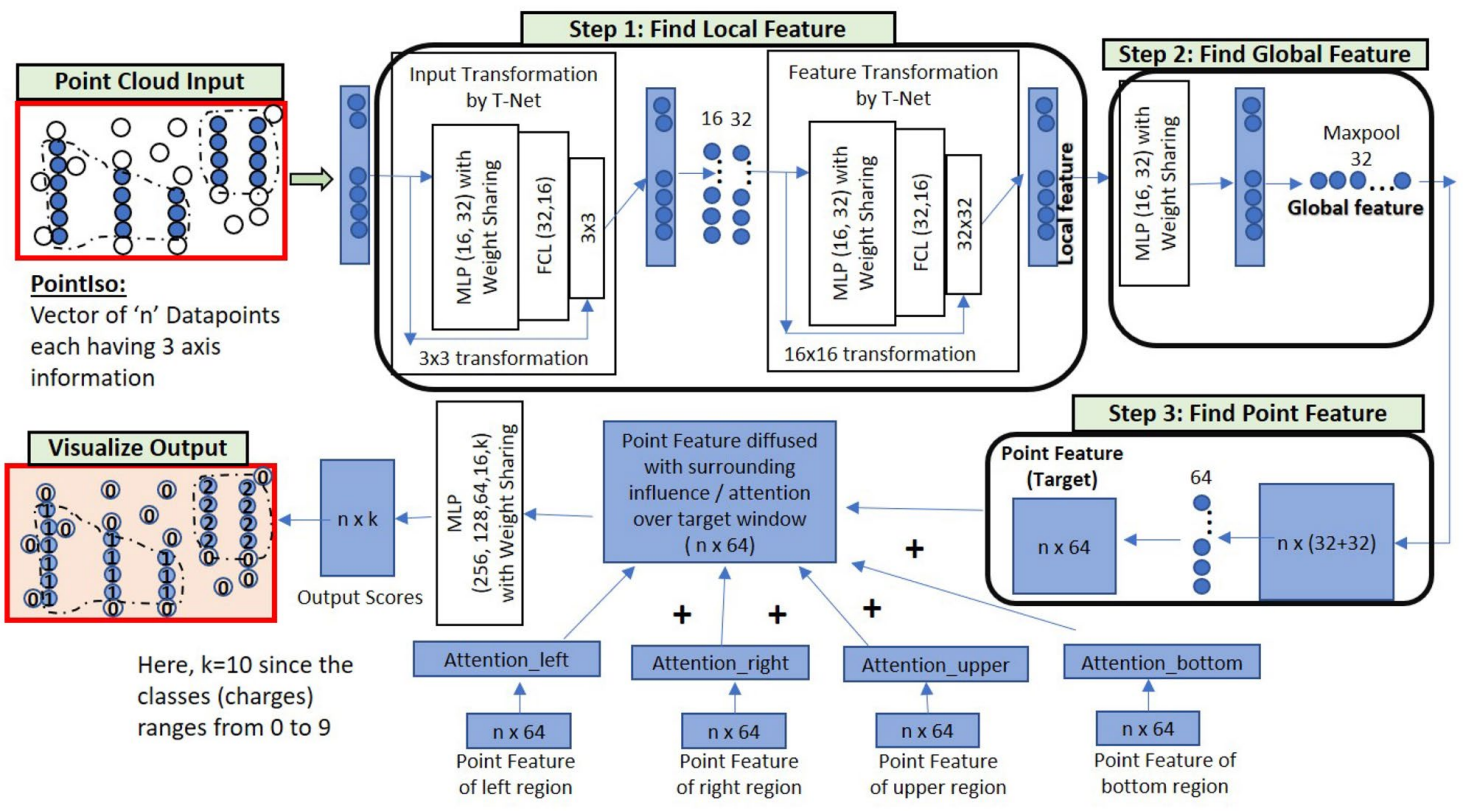
The network of IsoDetecting module. This network goes through three steps, finding the local features, global features, and point features respectively of the given target window. The number of layers and neurons in the Multiple Parceptron Layers (MLP) and Fully Connected Layers (FCL) is determined by experiments and mentioned in the figure. Point features of the target window are then diffused with features of surrounding regions based on their attention or influence over the target window (calculation of $Attention\_left$ and others are shown in the next figure). Finally, the diffused features are passed through four MLP, and the Softmax layer at the output provides the final segmentation result.

### Step 1: Scanning of LC-MS map by IsoDetecting module to detect isotopes

**Figure 7:**
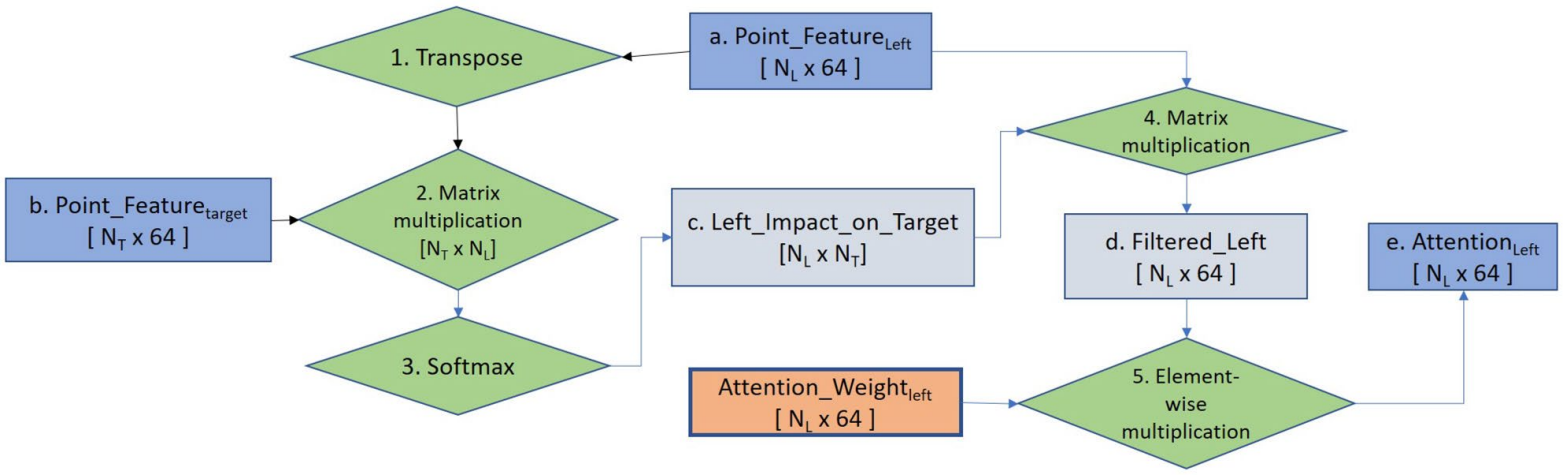
Flowchart of attention calculation in the IsoDetecting module. This particular flowchart is intended to find out the attention or impact of the left region over the datapoints of the target window. Exactly similar approach is followed for other surrounding regions as well and finally, all are diffused with the $Point\_Feature_{target}$ by addition.

Our network scans the 3D LC-MS plot using a non-overlapping sliding window having dimension 2.0 *m*/*z*, 15 RT scan, and covering full intensity range, as already presented in Fig. 1. The intensities are real numbers scaled between 0 to 255. Here the objects, i.e., peptide features are to be separated from the background. The background may contain feature-like noisy signals and peptide features are frequently overlapped with each other. So the target is to label each datapoint represented by a triplet (RT, *m*/*z*, intensity) with its class. The class is either charge z = 1 to 9 (positive) if the datapoint belongs to a feature having that charge, or $z=0$ if that datapoint comes from background or noise. Each window sees a point cloud which is essentially a set of points, or triplets (RT, *m*/*z*, intensity). This is passed through a PointNet architecture as shown in Fig. 6. In order to properly segment peptide features spreading over multiple sliding windows, we adapt the DANet^13^ and plug into our model to find the attention or influence of four surrounding regions (Fig. 4b) over the target window datapoints. We present a flowchart in Fig. 7, showing the calculation of attention coming from the surrounding regions. The detailed explanation of this flowchart is provided in Supplementary Method A.

We can divide the LC-MS map along *m*/*z* axis into sections of equal ranges and process multiple sections in parallel to make the process time-efficient. We keep nine hash tables for recording the detection coordinates (RT, *m*/*z*) of features from nine classes ($z=$ 1–9) during the scanning. The *m*/*z* values of the isotopes are used as the key of these hash tables, and the RT ranges of the isotopes in a feature are inserted as values under these keys as shown in the block diagram of Fig. 1. Since the detection of wider isotopes may span over a range of points along *m*/*z* axis, we take their weighted average to select specific *m*/*z* of an isotope.

**Figure 8:**
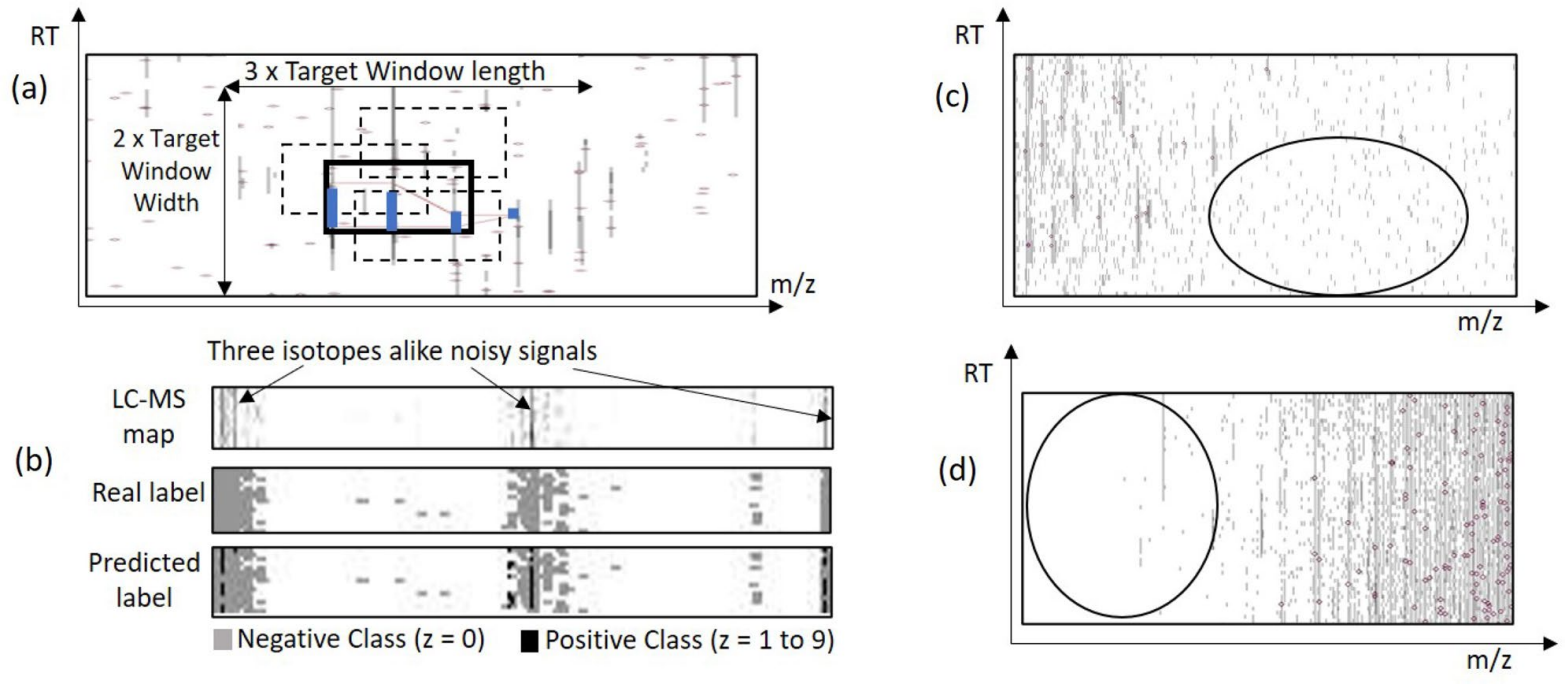
(**a**) An area in LC-MS map is shown. A target window in bold black rectangle containing a feature in blue color is shown. This target window and its four surrounding regions (as shown in Fig. 4b) forms a positive training sample for IsoDetecting module. We also slide the target window within the region showed by arrow signs to generate training samples holding the peptide feature in different locations of the target widow (some are shown by dotted rectangles). (**b**) One negative training sample containing feature like noises is shown the topmost rectangle. In this region of LC-MS map the traces look like a feature having three isotopes. However, those are actually noisy signals as shown in the middle rectangle. And if we do not provide such training samples, then PointIso label the respective datapoints as positive class and report it as a feature, as shown in bottom rectangle. That is why we should provide such samples during training. (**c**) Some region in LC-MS map containing only arbitrary noises is selected for generating negative training samples. (**d**) Some region in LC-MS map containing blank area is also selected for generating negative training samples.

**Table 4:** Class distribution of peptide features in our dataset consisting of 57 LC-MS maps.

Class (charge state)	1	2	3	4	5	6	7	8	9
Peptide features	163,038	863,050	428,909	29,183	1503	653	179	236	233

#### Training data generation for IsoDetecting module

IsoDetecting module is supposed to learn some basic properties of peptide feature, e.g., bell-shaped signal, equidistant isotopes, etc. (more are provided in Supplementary Note D), and many other hidden characteristics. From each LC-MS map, we cut out the peptide features for training, which we call *training samples*. Each training sample consists of a target window (which is called the sliding scanning window in the testing or application phase) and its four surrounding regions. The datapoints in the target window are labeled with a value within the range z = 0–9. Here, z = 0 means the respective datapoint belongs to noise or background, and z = 1 to 9 means the respective datapoint belongs to a feature having charge z. We slide the target window over the peptide feature and its surrounding region to mimic the testing scenario so that we can generate training samples holding peptide features in different locations of the target window, as shown in Fig. 8a. As already mentioned, we select the common list of peptide features provided by MaxQuant and Dinosaurs, to generate the positive training samples. The total number of such common peptide features available from each charge state is presented in Table 4. Next, we see the LC-MS maps in PEAKS Studio and we visually choose some regions containing noisy signals that look like features (Fig. 8b) (in our case it was around the retention time range 10 min), only noises of different intensities (Fig. 8c), and completely blank areas as well (Fig. 8d). We cut out some training samples from those regions (one target window and four surrounding regions as before) and call them negative training samples. Please note that all the datapoints in negative samples are labeled ‘0’. We see the total number of training samples in Table 5. Readers may wonder why we have included a column for ‘Total Datapoints’ in the table. Please note that, unlike DeepIso, the IsoDetecting module is a segmentation network here. As a result, it has to classify each datapoint in the target window of training samples. If a target window in a positive training sample prepared from peptide feature having charge ‘2’ contains features with other charges, e.g., ‘3’ and ‘4’, then the IsoDetecting module has to classify the datapoints belonging to not only charge ‘2’, but also charge ‘3’ and ‘4’. Therefore, the class sensitivity is impacted by the total amount of datapoints having that class, taking into account *all* the training samples. So we have included a column labeled as ‘Total Datapoints’ in Table 5. The positive and negative samples are generated from the validation LC-MS map similarly and presented in the table as well.

**Table 5:** Amount of samples for training and validation. Because of inadequate training data for features with charge states 5–9 as mentioned in Table 4, we had to apply data oversampling and augmentation to increase training samples from these classes. The amount of samples from class 0 depends on our choice. We chose the amount so that the total number of datapoints from this class is higher than others because the LC-MS map is very sparse. The validation set does not contain any duplicated data, and there is no overlapping between the validation dataset and the training dataset.

Class (*z*)	Training		Validation	
	Total datapoints	Total samples	Total datapoints	Total samples
0	544,014,927	40,502	22,100,416	10,138
1	3,648,512	37,924	167,671	1282
2	28,633,707	152,148	1,731,244	8902
3	19,021,011	91,542	1,033,477	4485
4	2,998,905	25,526	77,534	281
5	3,526,031	22,134	3032	13
6	431,139	7721	606	5
7	30,145	4472	319	4
8	18,144	8706	48	3
9	17,310	3429	227	2

#### Technical details on training and validation of IsoDetecting module

We use a minibatch size of 8 during the training because the network already takes about 15 GB GPU Memory due to the sophisticated architecture. We use ‘NAdam’ stochastic optimization^32^ with initial learning rate of 0.001. We check the accuracy on validation set after training on every 1200 training samples. If we do not observe any significant drop in the validation loss for about 5 epochs, we decrease the learning rate by half. The model converges within about 100 epochs. We use Sparse Softmax cross-entropy as the error function at the output layer. Besides that, we apply the class weights as already mentioned in the Discussion section and is elaborated in the Supplementary Information. The average sensitivity of the trained model on the training samples and validation samples are provided in Table 6 for fold 1.

**Table 6:** Class sensitivity of the IsoDetecting module for fold 1, i.e., dilution sample 10, 11, 12 are used for training and 9 is used for validation. We show two cases, average and best case in terms of feature detection ability. The best case occurs when the feature is left aligned with the target window boundary, e.g., feature ‘A’ in Fig. 4a. The average case means the target window might contain any number of features, they may appear at any location of the window, they might be partially or fully seen and might be overlapping as well. Due to the lack of variance in training data for charge states 6–9, the model’s validation sensitivity does not go up high for these classes. However, since most of the peptide features appear with charge states < 6, lower sensitivity for them does not impact the overall performance. The validation set does not contain any duplicated data and there is no overlapping between validation samples and training samples.

Class (*z*)	Training		Validation	
	Sensitivity-average (%)	Sensitivity-best (%)	Sensitivity-average (%)	Sensitivity-best (%)
0	88.48	30.0	88.48	38.69
1	57.59	91.0	61.54	99.28
2	76.90	97.0	82.98	98.40
3	75.08	94.75	79.42	96.05
4	64.78	94.24	52.80	97.33
5	88.57	94.21	58.74	100
6	60.73	82.5	15.68	70.56
7	40.12	60	10.03	70.78
8	4.07	10	3.0	10
9	4.50	8	2.01	15

**Figure 9:**
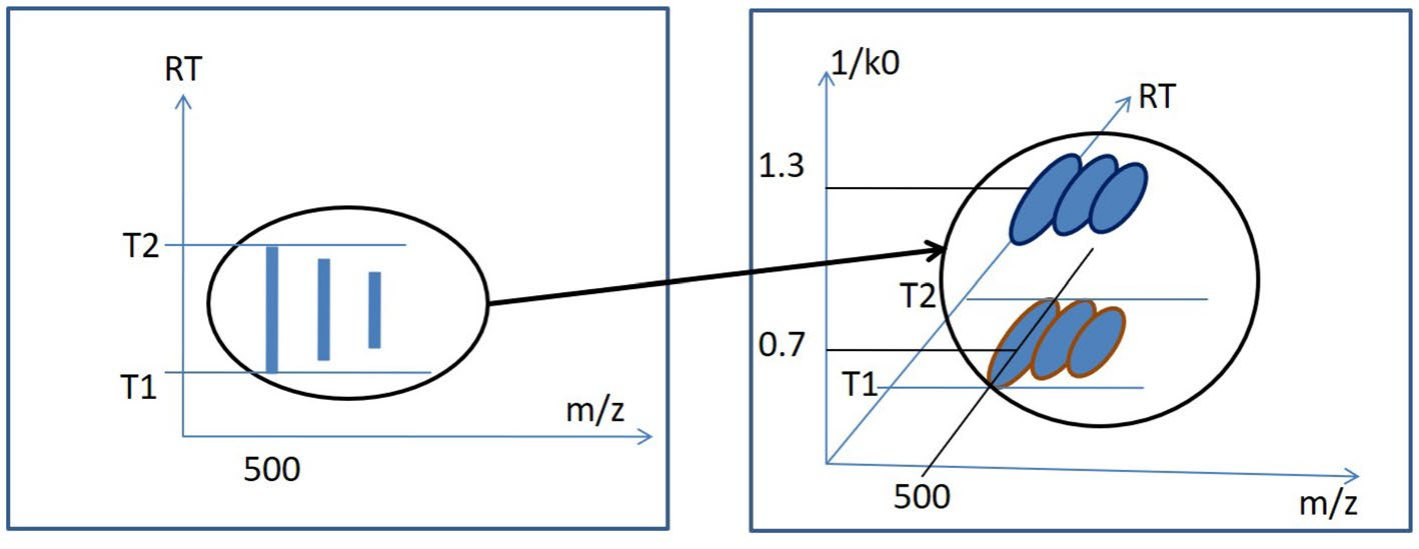
We show the peptide feature in $\frac{1}{k0}$ dimension. In this figure we omit the axis I for simplicity. In the left, we see an usual peptide feature extending along RT and *m*/*z* axis. This same feature can get separated into two different features if we consider the additional $\frac{1}{k0}$ dimension, as shown in the right.

#### Extension of IsoDetecting module for 4D data

We provide an illustration of TimsTOF generated 4D data in Fig. 9. It has $\frac{1}{k0}$ dimension additional to three other dimensions: *m*/*z*, RT, and I (we omit the axis I for simplicity in the figure). So the point cloud input consists of a vector of ‘n’ datapoints each having 4 axis information, *m*/*z*, $\frac{1}{k0}$, retention time, and intensity. To make the GPU matrix calculation manageable we reduce the number of neurons and layers in IsoDetecting module. In particular, following modifications are performed on the IsoDetecting network (please refer to Fig. 6):

- The last layer in the ‘Input Transformation by T-Net’ has now $4\times 4$ dimension since we are dealing with 4D data.
- There is only one layer having 16 neurons in between the ‘input transformation’ and ‘feature transformation’ networks.
- Last layer in ‘feature transformation’ network has $16 \times 16$ dimension.
- During finding the point feature, the concatenation of global feature and local feature gives $n \times (16+32)$ dimension since we are doing $16 \times 16$ feature transformation now.
- At the end, before the output layer, we keep three fully connected layers (256, 128, 64), and remove the last one having 16 neurons.

Besides bringing this changes to the network, we also modify the scanning window size to cover 1.0 *m*/*z* and 10 min RT. In this case, for the attention calculation network, we take the full region of left and right window. Because the features having charge 1 have isotopes 1.0 *m*/*z* distance apart. But for upper and bottom window we take 50% region as it seems enough for feature detection. We should mention that we had to reduce the network size due to shortage of GPU memory. Therefore, we encourage users to observe the performance of PointIso with 4D data keeping the original dimension if possible.

### Step 2: Scanning of LC-MS map by IsoGrouping module to report peptide feature

There are four major differences in IsoDetecting and IsoGrouping modules. First, the IsoDetecting module scans the LC-MS map along both the RT and *m*/*z* axis, whereas IsoGrouping module scans left to right, i.e., only along the *m*/*z* axis. Second, IsoDetecting network is a point cloud based network, whereas the IsoGrouping network is image-based. Third, the IsoGrouping module performs a sequence classification task that generates one output after seeing through 5 consecutive frames, unlike the IsoDetecting module which segments the datapoints of the input frame. Last, IsoDetecting module accepts very high resolution *m*/*z* values (up to 4 decimal place), but IsoGrouping works on comparatively lower resolution *m*/*z* values (up to 2 decimal place) because it does not need much higher resolution to group the isotopes into features.

**Figure 10:**
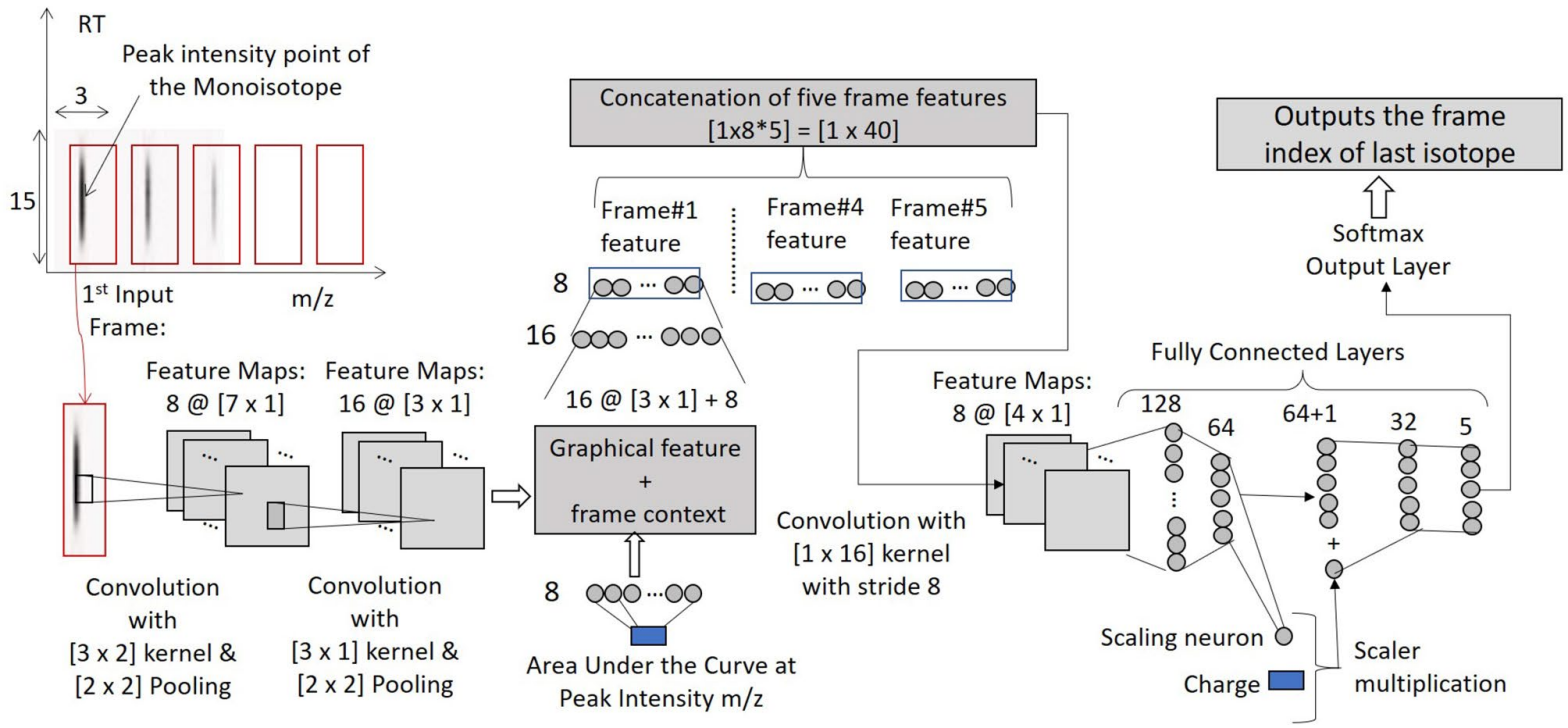
The network of IsoGrouping module. It starts with two convolution layers to fetch the graphical features from the input frame. Then we concatenate the intensity of the isotopic signal (area under the bell shaped isotopic signal) with it through an embedding layer of neurons (frame context). Then this is passed through two fully connected layers having sizes 16 and 8. This gives us the ‘frame feature’ of the input frame. We perform the same for five consecutive frames and then concatenate the ‘frame feature’ of those altogether. Then one layer of convolution is applied to detect the combined feature from all the frames. The resultant features are passed through two fully connected layers (size 128 and 64) to decide whether this is a noise or potential feature. This probability is also used to activate a scaling neuron, that feeds the charge into the network through proper scaling. The scaled charge is concatenated with the latest layer output (size 64) and passed through two fully connected layers. Finally, the Softmax output layer at the end classifies the sequence. We include pooling layers after the first and second convolution layers. We apply the ReLu activation function for the neurons. The dropout layers are included after each fully connected layer with a dropout probability of 0.5. The other network parameters are mentioned in the figure.

The IsoDetecting module provides us a list of isotopes. Equidistant isotopes having the same charge are grouped into a cluster or sequence. Then those sequences of isotopes are passed to the IsoGrouping module. Unlike DeepIso, we do not pass the isotopes directly to the second module, because here the input resolution is different in two modules. Therefore we apply a resolution degradation technique that filters out the region of isotope (as suggested by IsoDetecting module) from the background, present it in lower resolution, and then pass it to the IsoGrouping module. The detailed procedure is provided in the Supplementary Method B. We illustrate our proposed network for the IsoGrouping module in Fig. 10. A sequence of five frames is shown in top left of the figure (first three frames are holding isotopes and the rest two are blank). It may break each sequence into multiple features, or report one feature consisting of the whole sequence of isotopes, or even report that sequence as mere noise. It works on a sequence of isotopes in multiple rounds. Each round process five consecutive frames at a time. The output is i = 0 to 4, where, $i=0$ means that no feature starts at the first frame, so skip it. Output i = 1 to 4 means, there is a feature starting in the first frame, and it ends at $(i+1)$th frame. If output $i=4$, it means that the feature might have more than 5 isotopes. Those can be found by overlapping rounds. A step by step explanation of the scanning procedure with the figure is provided in Supplementary Method C.

**Figure 11:**
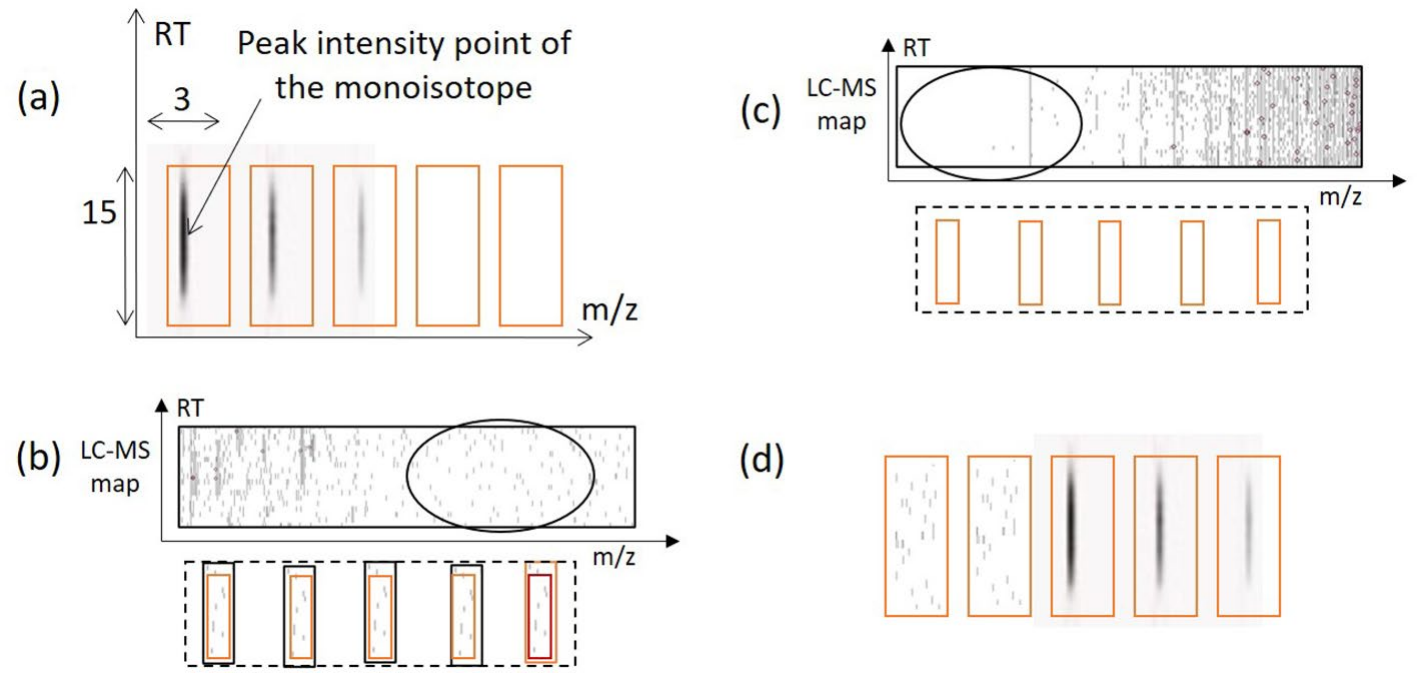
(**a**) A sequence of five frames, first three holding three isotopes. Each frame has dimension $[15 \times 3]$, covering 15 scans along the RT axis and 0.03 *m*/*z* along the *m*/*z* axis (each pixel represents 0.01 *m*/*z*). We filter out isotopic signals from the background by taking the intensity within the range of 2 ppm before and after the peak intensity *m*/*z* value, and 7 scans before and after the peak intensity RT value. The signal is left aligned with the frame. (**b**) A sequence of five frames which is generated from noisy areas in LC-MS map. (**c**) A sequence of five frames which is generated from blank areas in LC-MS map. (**d**) A sequence of five frames where the initial frames are holding noisy traces.

#### Training data generation for IsoGrouping module

Usually, monoisotope’s intensity is the highest among the other isotopes in a feature and dominates the total intensity of the feature. This property should be learned by the IsoGrouping module. We prepare positive training samples by generating a sequence of 5 frames for each peptide feature in the LC-MS maps, where the sequence starts at the first isotope of the respective feature, as shown in Fig. 11a. Each sequence is labeled by the frame index holding the last isotope of the feature (indexing starts from 0). So the sample in Fig. 11a has label 2. The minimum number of isotopes in a feature is 2, i.e., the minimum label of a positive sample can be 1. If the feature has equal to or more than 5 isotopes, the label is 4. We generate negative samples by cutting some sequences from the noisy (Fig. 11b) and blank area (Fig. 11c) and labeling them as 0. We generate sequences that contain peptide feature, but the feature does not start at the first frame of the sequence (Fig. 11d). Those samples are labeled as 0 as well, indicating that no feature starts at the first frame. We do this to handle the cases where noisy traces are classified as isotopes by the IsoDetecting module by mistake and thus clustered with the actual features in the intermediate step.

#### Technical details on training and validation of IsoGrouping module

For the training, we set minibatch size 128 and apply ‘NAdam’ stochastic optimization^32^ with initial learning rate of 0.001. We run the training for 10 epochs, with validation step run in every 1200 sample training. We use Softmax cross-entropy as an error function at the output layer. We see the training and validation sensitivity in Table 7. We observe that the maximum sensitivity of the classes > 0 is about 61% on the training set and about 64% on the validation set. To have a better perception we present the confusion matrix in Table 8. We see the model hardly misses the monoisotopes (low percentage of features misclassified as class A), but confuses about the last isotope of a peptide feature. Please note that reporting the monoisotope along with the first few isotopes (having higher intensity peaks) of a feature is more important in the workflow. Because they dominate the feature intensity and are used in the next steps of protein quantification and identification.

**Table 7:** Class sensitivity of the IsoGrouping module on the training set and validation set. The output is i = 0 to 4, where $i=0$ means that no feature starts in the first frame, so skip it. Output i = 1 to 4 means, there is a feature starting in the first frame, and it ends at $(i+1)$th frame. When output $i=4$, it means there might be more isotopes left. So we run another round of processing over the rest of the isotopes of the same cluster or sequence. Therefore, although our network process five frames at a time, if the feature has more than five isotopes, those can be found by overlapping rounds.

Class	Sensitivity on training set (%)	Sensitivity on validation set (%)
0 (noise)	89.42	90.78
1 (2 isotopes)	57.93	57.50
2 (3 isotopes)	51.98	43.30
3 (4 isotopes)	61.90	59.86
4 (5 isotopes or more)	61.77	64.14

**Table 8:** Confusion matrix for the prediction of IsoGrouping module on the validation dataset. The diagonal values, e.g. [2, 2] represent the sensitivity for class 2. We say a feature is misclassified as class 0 when the monoisotope (first isotope) or all of the isotopes are missed, i.e., the feature is thought to be noise by mistake. The value of [2, 0] indicates what percentage of features with three isotopes are either misclassified as noise, or monoisotope is missed. [2, 1] indicates the percentage of features which have three isotopes but the third one is missed, and only the first two are combined. Similarly [2, 3] shows for what percentage of three isotope features, IsoGrouping module finds ONE additional isotope at the end.

Class	0	1	2	3	4
0	89.43%	6.88%	1.72%	0.94%	1.03%
1	17.29%	57.93%	18.18%	5.58%	1.03%
2	5.38%	18.94%	51.98%	21.58%	2.13%
3	3.25%	5.44%	14.29%	61.90%	15.12%
4	5.71%	2.55%	3.18%	26.79%	61.77%

#### Extension of IsoGrouping module for 4D data

While sending the cluster of isotopes found from IsoDetecting to the IsoGrouping module, we actually compress the scans along the ion mobility, i.e., $\frac{1}{k0}$ dimension and two dimensional frames are passed to the IsoGrouping module. We do this to keep the changes minimal. After we get a final list of peptide features, we insert an additional $\frac{1}{k0}$ finding step. In this step, each feature is analysed along $\frac{1}{k0}$ axis. We know that each feature has a elution time range or retention time (RT) range. At each point of that range along RT axis, we have a scan $\frac{1}{k0} \times m/z$, that is bounded by the *m*/*z* range found from IsoDetecting and Isogrouping module. So within that bound, we look for group of equidistant vertical traces that presents the ion mobility signal. Then those groups are merged over different RT points within the range and we get a complete set of peptide features having *m*/*z*, $\frac{1}{k0}$, and RT values listed. Separate script for this additional $\frac{1}{k0}$ finding step is uploaded in Github repository mentioned in Data Availability section.

Finally, we would like to mention some common strategies followed for implementing and training both of the modules. We implemented our deep learning model in Python^33,34^ using the Google developed Tensorflow^35^ library. We check the accuracy on validation set after training on every 1200 samples. We continue training until no progress is seen on validation set for about 15 epochs. We perform data shuffling after each epoch which helps to achieve convergence faster. In the initial phase of project development, we used batch normalization layers in PointIso. It made the model converge quickly during training but does not improve the class sensitivity. Moreover, adding the batch normalization layer in IsoDetecting module took more GPU memory. That is why we discard it later to make the testing or application phase more memory efficient. For developing the PointIso we use Intel(R) Xeon(R) Gold 6134 CPU, NVIDIA Tesla GPU, and Ubuntu 16.04.5 LTS operating system.

## Supplementary Information

Supplementary Information.

## Data Availability

The benchmark 3D dataset is available to download from ProteomeXchange using accession number PXD001091, and 4D dataset from accession number PXD010012. The full experimental result on all the replicates of the samples is available in supplementary materials. Source code can be found at this address: https://github.com/anne04/PointIso.
