# Supplementary material for: Deep neural network for detecting arbitrary precision peptide features through attention based segmentation
Source: Sci Rep. 2021 Sep 14;11:18249. doi: 10.1038/s41598-021-97669-7 (PMC8440683; doi:10.1038/s41598-021-97669-7)
Supplement: Supplementary file 1 — Supplementary Information. [file 41598_2021_97669_MOESM1_ESM.pdf]

Deep Neural Network for Detecting Arbitrary Precision Peptide Features through Attention Based Segmentation

Fatema Tuz Zohora, M Ziaur Rahman, Ngoc Hieu Tran, Lei Xin, Baozhen Shan, Ming Li

Supplementary Note A

Experiment Category	z=0	z=1	z=2	z=3	z=4
Without any class weight (after 10 epochs)	96%	40%	53%	33%	15%
Class weight based on distribution over whole dataset (after 10 epochs)	91%	48%	68%	51%	27%
Class weight based on distribution over sample (after 10 epochs)	86%	51%	71%	55%	30%
Same as above, after convergence	64%	59%	79%	67%	14%
Same as above, with attention mechanism	50%	79%	95%	91%	85%
Same as above, with attention mechanism and higher resolution	40%	99%	98%	98%	98%

Supplementary Table S 1. Class sensitivity for different weighting mechanisms. We compare the candidate weighting mechanisms based on the sensitivity for the best case scenario, i.e., when feature is aligned with the left boundary of the scanning window (e.g., feature A in Figure 4(a)), with high abundant features, i.e., features having charge, z = 1, 2, 3, 4. Please note that, although the sensitivity of negative class (z=0) is comparatively lower for our chosen criteria, however, it does not imply that it reports many false positives. Although the datapoints which are very close or adjacent to the real signal, are sometimes predicted as positive points, but in general the negative class has higher class sensitivity than all others as presented in Table 7 of the main manuscript.

Class Weight Assignment Procedure

We choose the class weight based on distribution per sample. Let us have a sample window which contains 100 datapoints. They come from three different classes (z): 0, 2, and 3. We have 10 points from $z = 3$, 30 points from $z = 2$, and remaining 60 points from $z = 0$. Then points from class 0, 2, and 3 get weights of 0.4, 0.7, and 0.9 respectively. We perform this for every sample. So every training sample has a weight list associated with it, which we pass to the network during cross entropy loss calculation.

Supplementary Note B

Attention Mechanism

We would like to discuss our experiments to correctly segment partially covered peptide features in a target window. We started with considering a bigger scanning window which will produce output for the center region. This caused three problems. First, we can apply back-propagation based on full bigger window during training time (number of input nodes equals to the number of output nodes), but use the center region only during testing time or prediction (output nodes corresponding to center region are used only). In this case model gets confused about the boundary region during training and cannot learn well. Second, during training time we can apply back-propagation based on center region only, therefore, number of input nodes is not equal to the number of output nodes anymore. This actually makes the architecture very complex. Because number of datapoints in the center region, and surrounding r1, r2, r3, r4 regions is never fixed. Although we consider a fixed number and do padding for simplification, but when number of input is not equal to the number of outputs, internal design gets quite complicated. Also the fact that, datapoints in r1, r2, r3, r4 need not to worry about each other, only have to focus on center region, may not be well learned by the model. We would not have any control over that. Finally, we also face technical issues since GPU memory exceeded (more than 16 GB) with bigger region. Because of these reasons we were unable to proceed with the design.

So we applied next simpler technique, sliding windows with 50% overlapping. The resultant class sensitivities are presented in the first row of Table S2. After passing the IsoDetecting output through IsoGrouping module, it finally produce only 65% feature detection as presented in the first row of Table 5. Therefore we had to use more sophisticated approach to address this problem.

In Figure 4 of main manuscript, we see that the regions r_1, r_2, r_3, and r_4 are actually playing the key role in detecting the traces inside target window. We empirically tested following three techniques for diffusing the surrounding information into current window, and the results are summarized in Table S2.

Experiment Category	z=0	z=1	z=2	z=3	z=4
Sliding window with 50% overlapping	86%	50%	72%	57%	36%
Skiplink inserted in above model	85%	56%	76%	66%	41%
Bi-directional 2D RNN	85%	51%	77%	67%	49%
**Attention mechanism**	84%	62%	85%	71%	50%
**Attention mechanism with higher resolution**	90%	64%	85%	81%	61%

Supplementary Table S 2. Different techniques of absorbing surrounding information and corresponding class sensitivity of IsoDetecting module. We define the class sensitivity of a scanning window as the number of datapoints from class z (0 to 9) detected correctly out of total number of datapoints in a scanning window. To evaluate candidate solutions we use the class sensitivity of high abundant features (charge $z=$ 1,2,3, and 4) in a *average case* scenario. Average case means the scanning window might contain any number of features, they may appear at any location of the window, they might be partially or fully seen, and might be overlapping as well. We see that the DANet inspired attention based mechanism works better than other techniques.

- First, we just calculated the global features of surrounding regions and diffuse them together by addition and concatenation with the global features of target window. Then we repeated the 50% overlapping technique, which did not bring any significant change. Using some skip links along with that brings little improvement as shown in the second row of the table.
- Then we used a bi-directional two dimensional RNN network to flow information from all direction into the target window. The corresponding class sensitivities are provided in the third row.
- Finally, we applied the attention mechanism proposed by DANet which works better than first two techniques, as reported in the fourth and fifth row. So we choose PointNet segmentation network combined with DANet to develop our IsoDetecting module.

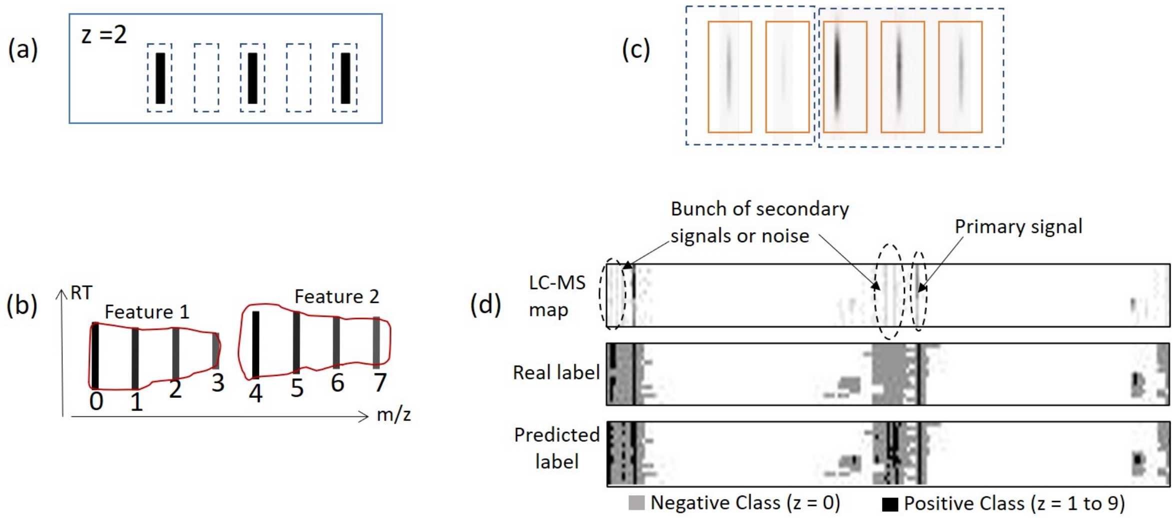

Supplementary Figure S 1. (a) Here we see a peptide feature with three isotopes and charge 2. But if this is passed to the IsoGrouping module with wrong frames (dotted rectangles) because of the wrong charge ($z=4$) predicted by the IsoDetecting step, it results in discarding this whole group of frames (by predicting it as noise) due to the inconsistency (blank frames) observed. (b) We see the adjacent feature problem. (c) We see one training sample prepared for IsoGrouping module to solve the adjacent feature problem. (d) In the topmost rectangle we see isotopes of a peptide feature in black traces and some secondary signals as well. The middle rectangle is showing the true labeling of the datapoints using two colors: grey means negative class and black means positive class. The primary signals are detected by PointIso as shown in the bottom rectangle. However, the seconadary signals are also predicted as positive class by PointIso. But this is wrong. Datapoints in those regions should be predicted as negative class by IsoDetecting module. So we select such peptide features for fine tuning.

Supplementary Note C

Fine Tuning Using Misclassified Features

In this section we will discuss the approach of fine tuning. Fine tuning the primary model by feeding back the misclassified data played an essential role in overall improvement. After we train and validate IsoDetecting and Isogrouping module, we run a full scanning on the same LC-MS maps used for training (e.g., in 3D dataset, LC-MS maps of dilution sample 9 to 12 for the first fold). That means, we scan the full LC-MS map by IsoDetecting module in a column by column order, which gives a list of sequence of equidistant isotopes. Then those are passed to IsoGrouping module for generating the final list of peptide features. Then this peptide feature list is matched against the MS/MS identified peptide features for the respective LC-MS map by MASCOT. Based on the matching result, we select the misclassified peptide features. By the term misclassified we mean four particular cases: peptide features not detected due to very long retention time range, peptide features detected with wrong charge, multiple peptide features grouped into one feature, and false positives due to secondary signals. Then we generate training samples from those misclassified cases as mentioned in the training data generation subsections of Method section in the main text and retrain both modules using those. We call this approach fine tuning by misclassified peptide features. After that we run the testing on test set. How do we detect those four types of misclassified features, extract those from LC-MS map, and label them for fine tuning PointIso, are explained below in more detail. While providing examples, we will be using LC-MS maps from fold 1.

- First, some identified peptide features were not detected and when we inspected those visually in the LC-MS map by PEAKS Studio, we discovered that those have comparatively longer retention time range, about 0.30 minute or longer. Please note that, those features were provided during initial training. But those cases are not learned well. So we provide such samples twice in the dataset (i.e., given more weight or emphasized by duplication) and retrain the model. It improves the detection rate by about 2% (fourth row in Table 3).

- Second, we compare the clusters or sequences of isotopes returned by IsoDetecting module with the MS/MS identification result for the respective LC-MS map and select those sequences which are matched with the identification result in terms of m/z range and retention time range but DOES NOT match in terms of charge and are also rejected later by IsoGrouping module. So such features are selected and fed back for further learning by IsoDetecting module. These sequences present the features which are detected by IsoDetecting module but with wrong charge. This usually happens when the feature appears in middle region of the target window (like 'F' in Figure 4(a) of the main manuscript). As a result, the wrong set of frames are passed to the IsoGrouping module, as shown in Figure S1(a). This causes complete rejection of the feature by the IsoGrouping module, and we miss a feature although it was detected in the first module. Please note that, it is possible that these features went through the usual training before. However, since these selected ones are not learned well, so we are just asking the IsoDetecting module to learn these again with greater emphasis (ensured by duplicating these samples during retraining as done in previous point). Retraining the IsoDetecting module using such samples improves the detection rate by about 1.5% (sixth row in Table 3 of main manuscript).

- Third, adjacent features as shown in Figure S1 (b) were not correctly separated by IsoGrouping module. Here, both features have the same charge, same or very close RT value at the peak intensity point, and the distance between the two features along m/z axis is equal to the distance between their own isotopes. We select such features by comparing the sequences of isotopes returned by IsoDetecting module with the MS/MS identified features for the respective LC-MS map and finding out those sequences where the $2^{nd}/3^{rd}/4^{th}/5^{th}$ frame matches with the identification result in terms of m/z, RT, and charge, but not the 1st frame. Because it means that the monoisotope appears on the $2^{nd}/3^{rd}/4^{th}/5^{th}$ frame and the isotopes preceding it are coming from some other feature that is preceding and adjacent to this matched feature. So we select such sequence and label them in a way which will break the feature into two. For example, the sequence shown in Figure S1(c) is labeled as 1. So that IsoGrouping will learn to output '1' for this sequence and this will break it into two features as shown by the dotted rectangles.

- Finally, we fine tune the model with false positives which are essentially some feature-like noisy or secondary signals appearing very close to the main isotopic signal (Figure S1(d), topmost rectangle). Our initial trained model reports those feature-like signals as peptide features (Figure S1(d), bottom rectangle) and as a result we get about 200,000 peptide features in total. We visually traced some of the false positive features and realized that those are actually secondary signals. Although random noise removal is learned easily by PointIso but separation of feature like noisy traces and those secondary peaks removal is difficult for PointIso. This tasks has to be performed by IsoDetecting module since it has access to the whole context. So it has to see through all the signals and decide which ones are to be reported and which are to be ignored. In order to collect those wrong reports, we match the peptide feature list returned by PointIso (for the 4th replicate of dilution sample 9, in fold 1) with the peptide feature list produced by all other tools (i.e., OpenMS,

MaxQuant, Dinosaurs, and PEAKS) with an error tolerance of $0.01 m/z$ and 0.2 min RT. The features from PointIso which neither match with other tools nor with the identification result by MASCOT are selected for fine tuning (about 70,000 features). As mentioned in the Method section, a training sample for the IsoDetecting module consists of a target window and its four surrounding regions. So we cut out those false positive features from the respective LC-MS map keeping the feature in the target window. All the datapoints in the target window which do not belong to primary signal are labeled as '0'. Then these newly generated training samples (about 30,000) are used for retraining the already trained model, by running few epochs with 0.0001 learning rate and keeping the best model state. This same set of features is also used for fine tuning IsoGrouping module. In order to make a training sample for fine tuning IsoGrouping module, we pick a false positive (isotopes are secondary signals) and cut a sequence of 5 frames each holding the respective secondary signals. Then we label the sequence as '0' indicating that no feature exists in that sequence. We can actually balance between the true positive detection rate and false positive detection rate using the amount of such training samples. Without any fine tuning we get 99.50% detection, with about 200,000 total features. After fine tuning with 30,000 samples we have 98% detection with 100,000 features (which is reported in the main result). If we fine tune with 50,000 samples then total number of features drop to 80,000 but the detection percentage also falls to 97.53%. So it seems like if we are too strict about discarding the secondary signals, we may also miss some true lower intensity peptide features which are close to other higher intensity peptide features. It should be an interesting research scope to see if we can solve this problem without a reduction in detection percentage. Besides that, we believe the necessity of this step also depends on the dataset and the mass spectrometer used. Because high-resolution mass spectrometers usually produce narrower signals without those secondary peaks, thus we might avoid this step and obtain over 99% detection. In our manuscript we have used fine tuned model of IsoDetecting module. But the other models are also uploaded in the GitHub repository. Users can choose according to their need. They can also fine tune further if necessary.

Supplementary Note D

Basic Properties of Peptide Feature

It is supposed to learn following basic properties of peptide feature, besides many other hidden characteristics from the training data.

1. In the LC-MS map, the isotopes in a peptide feature are equidistant along m/z axis. For charge $z = 1$ to 9, the isotopes are respectively 1.00 m/z, 0.5 m/z, 0.33 m/z, 0.25 m/z, 0.17 m/z, 0.14 m/z, 0.13 m/z, and 0.19 m/z distance apart from each other[?].

2. The intensities of the isotopes form bell shape within their retention time (RT) range as shown in the zoomed in view of Figure 1 of the main manuscript.

3. Peptide features often overlap with each other.

Supplementary Note E

Cross-Validation Technique

Our model is trained and evaluated through 2-fold cross-validation. That is, we divide the LC-MS maps in the dataset into two groups or two folds. Then we train on one group and test on the other group, and vice versa. In order to save the best model state during training, we keep one LC-MS map from the training set as a validation set. Then we use that model state for testing. Whenever we say train or validation on a LC-MS map, we mean training/validation on the features cut from that LC-MS map (explained later under the subsections regarding training data generation for IsoDetecting and IsoGrouping module). However, when we say test on a LC-MS map, we actually mean scanning the full LC-MS map as shown in the block diagram of Figure 1 in main text. That is, during testing (or the real application phase) we will actually scan the whole LC-MS map in a bottom up, left to right fashion (in other words, column by column). Now, we discuss how the LC-MS maps are divided into different folds for cross-validation as follows:

- In the 3D dataset (downloaded from ProteomeXchange with accession number PXD001091), there are 12 dilution samples for the LC-MS analysis, each sample having a different concentration of spike proteins. Each sample goes through the physical LC-MS instrument and produce a LC-MS map. Usually each experiment is repeated multiple times, and each resultant LC-MS map is called a replicate. Dilution sample 1 and sample 5 to 12 have 4 replicates each (e.g., 130124_dilA_1_01, 130124_dilA_1_02, 130124_dilA_1_03, 130124_dilA_1_04 are the four replicates for sample 1. These names are also mentioned in the final result shown in Supplementary Table S3). Dilution sample 2, 3, and 4 have 7 replicates each. Therefore, we have 57 LC-MS maps in total. We divide these samples or LC-MS maps into 2 groups, X

and Y. Group X contains the LC-MS maps from sample 9, 10, 11, 12. Group Y contains the LC-MS maps from sample 1 to 8. We first train the model on Group X (i.e., sample 10,11,12 are used for training and sample 9 is used as a validation set to save the best state of the model) and test the trained model on Group Y. Then we do the opposite. We train the model on Group Y (sample 5, 6, 7, 8 are used for training and sample 1 is used for validation) and test the trained model on Group X. The result of testing on all the samples is provided in Supplementary Table S3. Please note that, we report the result separately for each replicate. Detection percentage for a sample is the average detection percentage for all the replicates of that sample. We have already reported the detection percentage for 12 samples in Figure 2(a) and Table 1 of main text.

- Next, we discuss the distribution of LC-MS maps in our 4D dataset (downloaded from ProteomeXchange with accession number PXD010012) into different folds. For the 4D dataset, we have samples from two mobile phases: A and B. A provides 12 LC-MS maps (denoted as 2042 to 2053) and B provides 4 LC-MS maps (2054 to 2057). In total we have 16 LC-MS maps. Therefore we just divide it into two groups having equal size. Group X has 8 LC-MS maps: 2042 to 2049. Group Y has the remaining 8 LC-MS maps: 2050 to 2057. Just like before, we train on Group X (2044 is used for validation and the rest for training) and test on Group Y. Then we train on Group Y (2054 is used for validation and the rest for training) and test on Group X. The test result for each LC-MS map is provided in the plot shown in Figure 2(c) of main text.

Supplementary Method A

Attention Calculation

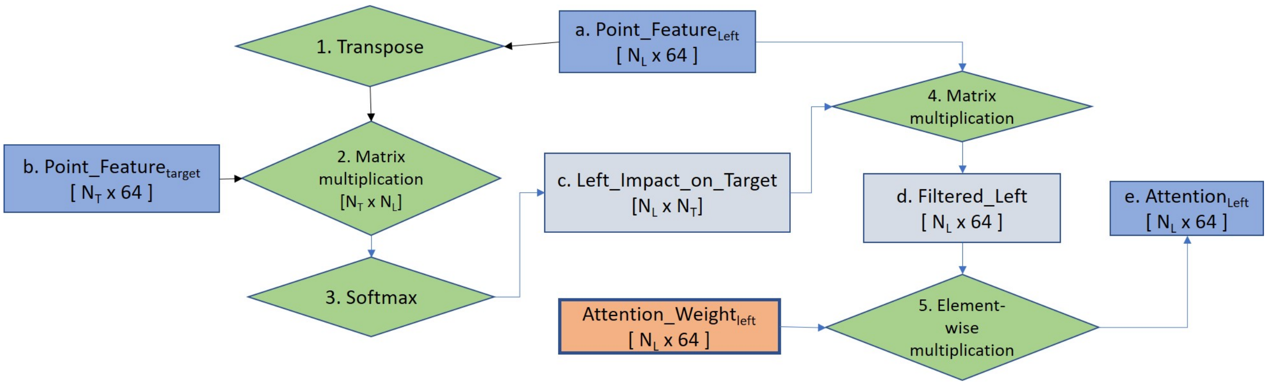

Supplementary Figure S 2. Flowchart of attention calculation in IsoDetecting module. This particular flowchart is intended to find out the attention or impact of left region over the datapoints of target window. Exactly similar approach is followed for other surrounding regions as well and finally all are diffused with the $Point\_Feature_{target}$ by addition.

Please refer to the flowchart shown in Figure 2. We have the point features of left region, $Point\_Feature_{left}$ according to the PointNet architecture shown in the main manuscript. Then we multiply $Point\_Feature_{target}$ with the transpose of $Point\_Feature_{left}$ according to the rule of matrix multiplication. Then we take softmax of the product as Equation 1, which gives us a $[N_T \times N_L]$ matrix, $Left\_Impact\_on\_Target$, where, N_T and N_L are the total number of datapoints of the target window and left region respectively. So each row presents a datapoint from target window and columns present the datapoints from left region.

$$Left\_Impact\_on\_Target = \frac{exp((Point\_Feature_{target_i}).(Point\_Feature_{left_j}))}{\sum_{i=1 \text{ to } N_L} exp((Point\_Feature_{target_i}).(Point\_Feature_{left_j}))} \quad (1)$$

Therefore, $Left\_Impact\_on_{Target_{(j,i)}}$ presents the attention of ith point of left region over the jth point of target window. The higher the value, the higher the correlation (similar feature) between those two points. So the jth row tells us which datapoints from left region have higher attention or highly correlated with the jth datapoint of target window. Next, we want to fetch those *significant point features from left region. So we apply another round of matrix multiplication (4th operation) between $Left\_Impact\_on\_Target$ and $Point\_Feature_{Left}$. We denote the resultant product as $Filtered\_Left$ since it essentially gives us $Point\_Features_{left}$ but scaled/filtered according to the aforementioned correlation or attention. Then again we have to know how much of those filtered features should be incorporated with the $Point\_Feature_{target}$ while segmenting the

datapoints of target window. So we use a weight matrices, namely $Attention\_Weight_{left}$, and multiply it with $Filtered\_Left$, producing $Attention_{left}$, which is finally passed forward to be diffused (by addition) with the $Point\_Feature_{target}$. This $Attention\_Weight_{left}$ is learned through training.

Supplementary Method B

Resolution Degradation in IsoGrouping Module

IsoDetecting module generates sequences of potential isotopes which are sent to the IsoGrouping module for final detection of peptide features. Now, the m/z value of isotopic signals are real numbers having up to 4 decimal places. We degrade each signal into real numbers having up to 2 decimal places. For example, let us have two sequences of isotopes A, and B, where the first isotope of the sequences are denoted as A_1 and B_1 respectively. The m/z values of these are respectively $A_{1\_mz} = 500.2351$ and $B_{1\_mz} = 500.2443$. During resolution degradation we filter out the signal instensity from the background with +-2 ppm m/z range. The range is calculated as: $\frac{A_{1\_mz} \times 2.0}{10^6}$. So that we have $A_{1\_mz} = 500.24$ and intensity is set as the maximum of the intensities of datapoints within range 500.2341 to 500.2361. Similarly, $B_{1\_mz} = 500.24$, *but* intensity is set as the maximum of the intensities of datapoints within range 500.2433 to 500.2453. So we see that, although both have the same m/z values in lower resolution, they definitely belong to different sequences and have different intensities (thus might have different pattern as well).

Supplementary Method C

Scanning Procedure by IsoGrouping Module

This method is intentionally kept similar to DeepIso. Although we have upgraded the internal architecture significantly, for the sake of user convenience we do not change the scanning procedure. Therefore, we request our readers to refer to the corresponding material from DeepIso.

Supplementary Table S3

Supplementary Table S3: Percentage of identified peptide features by different algorithms

LC-MS maps	OpenMS	MaxQuant	Dinosaurs	**PointIso**	Peaks
130124_dilA_1_01	94.2266	94.6575	95.4761	**98.0181**	95.6053
130124_dilA_1_02	96.0611	94.8955	95.4582	**98.8344**	95.8601
130124_dilA_1_03	95.9013	95.3994	95.8176	**98.2016**	96.0686
130124_dilA_1_04	95.444	95.1351	95.444	**98.1467**	95.5598
	95.40825	95.021875	95.548975	**98.3002**	95.7735
130124_dilA_2_01	96.3771	95.2163	96.2012	**98.593**	95.7088
130124_dilA_2_02	95.6753	95.5335	96.1361	**98.3339**	95.6044
130124_dilA_2_03	96.107	94.9221	95.9039	**99.0521**	95.7346
130124_dilA_2_04	95.8291	94.9135	95.6256	**98.4401**	78.942
130124_dilA_2_05	96.0703	95.0362	95.6222	**98.1041**	95.5188
130124_dilA_2_06	95.7873	94.6133	95.8564	**98.308**	95.442
130124_dilA_2_07	96.2179	95.7668	95.975	**98.3692**	95.8362
	96.00914286	95.1431	95.90291429	**98.4572**	93.2553
130124_dilA_3_01	95.9933	95.3872	96.2963	**98.3838**	95.9933
130124_dilA_3_02	96.3699	95.6849	96.4041	**98.6301**	96.3014
130124_dilA_3_03	96.0927	95.596	96.8212	**98.543**	96.6225
130124_dilA_3_04	96.1678	95.0446	95.9035	**99.0089**	95.9035
130124_dilA_3_05	95.9974	95.7392	96.417	**98.5474**	96.3525
130124_dilA_3_06	96.5644	95.497	96.5977	**98.0654**	96.0974
130124_dilA_3_07	96.3929	95.5556	95.942	**98.0998**	96.1997
	96.22548571	95.50064286	96.34025714	**98.46834286**	96.21
130124_dilA_4_01	96.3514	95.6784	96.3868	**98.1226**	96.3868
130124_dilA_4_02	95.8527	95.3522	95.6382	**97.4973**	96.2817
130124_dilA_4_03	96.286	96.286	96.7025	**98.2645**	96.772
130124_dilA_4_04	96.011	95.4952	96.4237	**98.7276**	96.2173
130124_dilA_4_05	96.3086	95.5437	96.1756	**98.9691**	96.0758
130124_dilA_4_06	96.4737	95.5755	96.6401	**98.4365**	96.3407
130124_dilA_4_07	97.3072	96.0771	97.0412	**98.1383**	96.5426

	96.37008571	95.71544286	96.42972857	**98.30798571**	96.3738
130124_dilA_5_01	95.6699	95.6699	96.151	**97.9645**	96.225
130124_dilA_5_02	95.747	95.8899	96.2831	**98.0343**	96.1401
130124_dilA_5_03	96.3406	95.3488	96.3748	**97.777**	96.4774
130124_dilA_5_04	96.1288	94.9298	96.026	**98.9723**	95.9918
	95.971575	95.4596	96.208725	**98.187025**	96.2086
130124_dilA_6_01	96.4139	96.1524	96.6007	**97.5719**	96.526
130124_dilA_6_02	96.0598	95.3667	96.1328	**98.5042**	96.1693
130124_dilA_6_03	96.3015	95.28	95.8084	**98.3093**	95.7027
130124_dilA_6_04	95.321	95.2186	95.8333	**97.4044**	95.4918
	96.02405	95.504425	96.0938	**97.94745**	95.9724
130124_dilA_7_01	95.4466	94.7167	95.4814	**98.1578**	95.1338
130124_dilA_7_02	96.1326	95.9254	96.6851	**98.9986**	95.9945
130124_dilA_7_03	95.9586	95.0568	96.0254	**98.664**	95.992
130124_dilA_7_04	95.7404	94.929	95.9094	**97.7688**	95.6051
	95.81955	95.156975	96.025325	**98.3973**	95.6814
130124_dilA_8_01	95.9857	95.4122	96.0573	**97.957**	95.6631
130124_dilA_8_02	95.8304	94.7703	95.4417	**97.7739**	95.689
130124_dilA_8_03	95.9361	95.3803	96.3876	**98.2633**	96.1445
130124_dilA_8_04	95.7613	95.3204	96.0665	**98.2028**	95.9986
	95.878375	95.2208	95.988275	**98.04925**	95.8738
130124_dilA_9_01	95.604	94.8282	95.9365	**98.7071**	95.4932
130124_dilA_9_02	96.3676	95.5685	96.6945	**98.1475**	96.5492
130124_dilA_9_03	95.5315	95.1712	96.036	**97.9459**	95.6396
130124_dilA_9_04	96.2108	95.453	96.3142	**98.5188**	95.8663
	95.928475	95.255225	96.2453	**98.329825**	95.8871
130124_dilA_10_01	95.3586	95.2436	96.0491	**98.3122**	95.8189
130124_dilA_10_02	94.8629	95.3418	96.5607	**96.8219**	96.3431
130124_dilA_10_03	95.7868	95.1026	95.7148	**97.8394**	95.9309
130124_dilA_10_04	96.4209	95.4903	96.3135	**98.5326**	96.4209
	95.6073	95.294575	96.159525	**97.876525**	96.1284

130124_dilA_11_01	96.0899	95.3411	95.9651	**98.4193**	95.7155
130124_dilA_11_02	95.2318	95.2759	96.0706	**95.9382**	95.4525
130124_dilA_11_03	95.5888	95.1311	95.4224	**97.0037**	95.5472
130124_dilA_11_04	95.5084	94.7325	95.0592	**98.1625**	95.3042
	95.604725	95.12015	95.629325	**97.380925**	95.5049
130124_dilA_12_01	95.0474	94.3625	95.2582	**95.8377**	94.8894
130124_dilA_12_02	95.7128	94.7481	95.7128	**95.9271**	94.8553
130124_dilA_12_03	95.7011	94.5752	95.6499	**96.4176**	95.5476
130124_dilA_12_04	95.3646	94.4271	95.4167	**97.3958**	95
	95.456475	94.528225	95.5094	**96.39455**	95.0731
1	95.40825	95.021875	95.548975	**98.3002**	95.7735
2	96.00914286	95.1431	95.90291429	**98.4572**	93.2553
3	96.22548571	95.50064286	96.34025714	**98.46834286**	96.21
4	96.37008571	95.71544286	96.42972857	**98.30798571**	96.3738
5	95.971575	95.4596	96.208725	**98.187025**	96.2086
6	96.02405	95.504425	96.0938	**97.94745**	95.9724
7	95.81955	95.156975	96.025325	**98.3973**	95.6814
8	95.878375	95.2208	95.988275	**98.04925**	95.8738
9	95.928475	95.255225	96.2453	**98.329825**	95.8871
10	95.6073	95.294575	96.159525	**97.876525**	96.1284
11	95.604725	95.12015	95.629325	**97.380925**	95.5049
12	95.456475	94.528225	95.5094	**96.39455**	95.0731
average	95.85862411	95.24341964	96.00679583	**98.00804821**	95.66185833

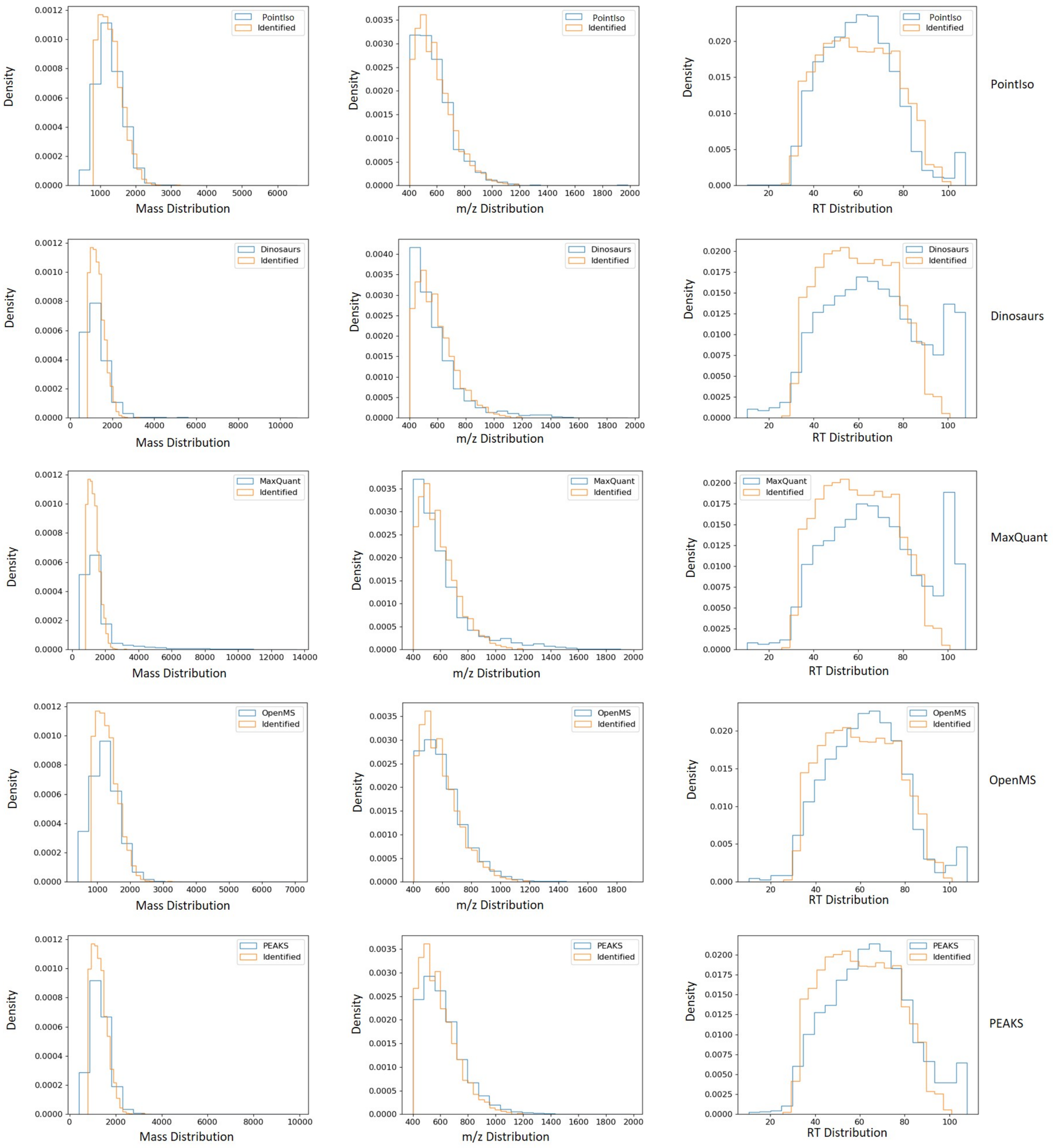

Supplementary Figure S 3. Comparison of mass, m/z and RT distribution of detected features (blue) and identified features (orange) for different tools.

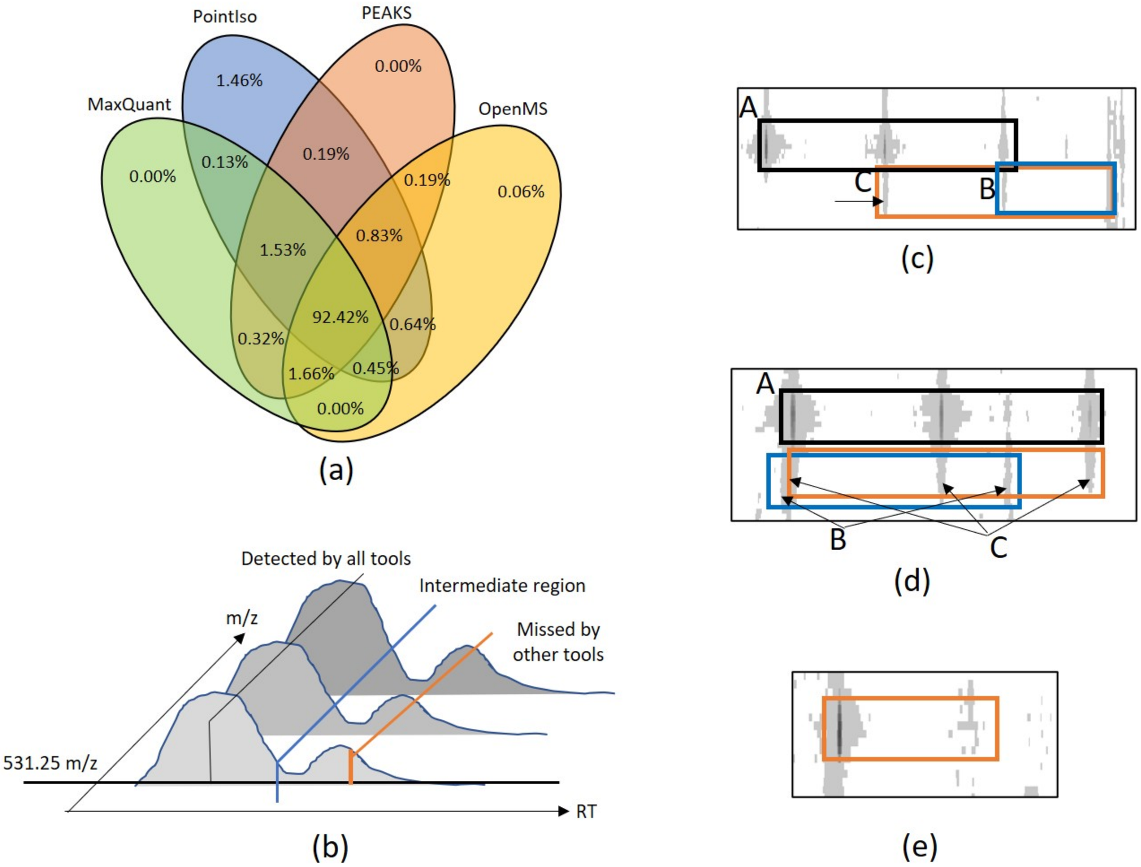

Supplementary Figure S 4. Observations on identified peptide features detected by only PointIso. (a) Venn diagram of identified peptide features detected by different algorithms (we show four algorithms to keep the Venn diagram simple). (b) Peaks connected by the black line are detected as a peptide feature by all algorithms. However, the feature having a lower peak, connected by the orange line, is missed by other tools. Because they have the same m/z as the one with a higher peak. Therefore, most of the tools merge it with the bigger one during pre-processing steps. (c) Monoisotope of the feature enclosed in the orange rectangle is missed by other tools due to merging with A. Here, feature A and C are detected by PointIso. But other tools detect A, and instead of C, they report B by mistake, missing the monoisotope (merging it with A). (d) Very closely residing and overlapping features, like feature B in blue and C in orange rectangles are sometimes missed by other tools as well, although detected by PointIso. (e) Feature with broken signals are detected by our model, but discarded by other algorithms.
